# EGCG and Taurine Synergistically Ameliorate Lipid Metabolism Disorder by Modulating Gut Microbiota and PPARα/FAS Signaling Pathway

**DOI:** 10.3390/nu17162595

**Published:** 2025-08-09

**Authors:** Yang Xiao, Mingyue Yang, Meihong Cai, Haihui Zhang, Kai Hu, Yuqing Duan

**Affiliations:** 1 School of Food and Biological Engineering, Jiangsu University, Zhenjiang 212013, China; 2222218045@stmail.ujs.edu.cn (Y.X.); 19802591983@163.com (M.Y.); zhanghh@ujs.edu.cn (H.Z.); kai.hu@ujs.edu.cn (K.H.); 2 Institute of Food Physical Processing, Jiangsu University, Zhenjiang 212013, China

**Keywords:** EGCG, taurine, lipid metabolism abnormalities, gut microbiota, metabolomics, PPARα/FAS

## Abstract

Background/Objectives: The synergistic effects of epigallocatechin gallate (EGCG) and taurine in modulating lipid metabolism abnormalities in rats were investigated, and along with their potential mechanisms. Methods/Result: Compared to intervention with EGCG/taurine alone, EGCG combined with taurine (1:3) not only reduced triglyceride (TG) generation in HepG2 cells (46.2%, 75.2%, respectively), but also significantly decreased the total cholesterol (TC) (33.3%, 41.8%), low-density lipoprotein cholesterol (LDL-C) (32.3%, 29.2%) in rats, while the high-density lipoprotein cholesterol (HDL-C) increased by 12.7% and 33.5%. In addition, the combination of EGCG and taurine not only inhibited lipogenic enzyme activity, but also enhanced the levels of lipid catabolic enzymes and antioxidant enzymes, and alleviated hepatic injury. Furthermore, it significantly modulated gut microbiota composition by altering the abundances of Bacteroidetes, Firmicutes, and Proteobacteria, improving intestinal flora balance. Metabolomic profiling showed that reducing N-linoleoyl proline, cortisol, and 3-isocholanolic acid, and increasing phospholipid metabolites are the main ways methods for normalizing lipid metabolism in rats. The combination also elevated short-chain fatty acid (SCFA) synthesis, preserving intestinal barrier integrity; it also promoted lipid catabolism and energy expenditure via activating Peroxisome proliferator- activated receptor alpha (PPARα) and suppressing hepatic fatty acid synthase (FAS)- mediated lipogenesis. Conclusion: These findings indicated that EGCG and taurine can synergistically regulate lipid metabolism abnormalities, which may offer a strategy for regulating lipid metabolism anomalies.

## 1. Introduction

Since the early 21st century, the global prevalence of metabolic diseases has continued to rise with increasing mortality rates, among which complications arising from lipid metabolism disorders constitute a substantial proportion [1]. Lipid metabolism dysfunction is characterized by abnormal blood lipid regulation, excessive hepatic lipid accumulation, and gut microbiota dysbiosis, frequently associated with unhealthy dietary patterns such as high fat intake [2]. Evidence demonstrates that long-term consumption of high-fat and high-sugar diets not only directly impacts lipid metabolism but also disrupts intestinal microecological balance by altering gut microbiota composition and diversity, thereby exacerbating metabolic disorders. Recent studies reveal that lipid metabolism dysregulation can induce hepatic injury and compromise intestinal barrier function, leading to gut microbiota imbalance and metabolic homeostasis disruption. As the human “second genome,” the gut microbiota exhibits compositional and functional changes that are intimately linked to host lipid metabolism. Microbiota imbalance not only impairs the production of beneficial metabolites such as short-chain fatty acids (SCFAs) but also modulates hepatic lipid synthesis and catabolism through the gut–liver axis. These pathological alterations further aggravate lipid metabolism disorders and associated complications through positive feedback mechanisms [3]. Currently, statins represent the primary pharmacological intervention for lipid metabolism disorders; however, long-term administration may result in adverse effects including renal dysfunction and hepatic injury [4].

Recent studies have highlighted the significant potential of plant bioactive compounds in regulating lipid metabolism disorders [5,6]. As a widely consumed beverage with deep cultural roots in East Asia, tea is rich in epigallocatechin gallate (EGCG), the most representative polyphenol exhibiting multifaceted mechanisms for the amelioration of metabolic abnormalities, which can activate the AMP-activated protein kinase (AMPK) signaling pathway, enhance peripheral glucose uptake and suppress hepatic gluconeogenic enzyme phosphoenolpyruvate carboxykinase (PEPCK) expression [7]. Concurrently, EGCG inhibits hepatic fatty acid synthase (FAS) and acetyl-CoA carboxylase (ACC) activities [8,9], resulting in reduced lipid accumulation in adipocytes and diminished hepatocellular lipid droplet size. Moreover, EGCG modulates gut microbiota composition by increasing beneficial bacteria and decreasing potential pathogens, thereby improving gut–liver axis metabolic interactions through enhanced SCFAs production [10,11]. The antioxidant effects of EGCG mainly include upregulating superoxide dismutase (SOD) and glutathione peroxidase (GSH-Px) activities, effectively scavenging 2,2-Diphenyl-1-picrylhydrazyl (DPPH) radicals, and reducing malondialdehyde (MDA) formation, which collectively decreases apoptosis rates. Additionally, EGCG can promote adiponectin secretion to maintain adipose tissue functionality [12,13]. Further evidence demonstrates that EGCG regulates lipid metabolism by activating peroxisome proliferator-activated receptor alpha (PPARα) to enhance hepatic fatty acid oxidation, increasing lipoprotein lipase (LPL) activity, and suppressing the FAS pathway, thereby lowering serum free fatty acid (FFA) levels [14,15].

Taurine (2-aminoethanesulfonic acid, C_2_H_7_NO_3_S), an endogenous β-amino acid abundantly present in mammalian tissues, exerts diverse physiological functions including neuromodulation, cardiovascular protection, and immune regulation [16]. As early as the 1980s, taurine was first reported to reduce total cholesterol (TC) levels in serum and liver of diet-induced hypercholesterolemic rats [17], drawing sustained attention to its potent lipid-lowering effects [18]. Additionally, serving as a nutrient for beneficial microbes such as *Lactobacillus* and *Bifidobacterium*, taurine supports their growth, modulates intestinal pH, and suppresses potential pathogens including *Escherichia coli* and *Clostridium* species through nutrient competition, thereby promoting gut health and microbial balance [19]. Recent studies further demonstrate that taurine regulates multiple critical biological processes, such as inflammation, oxidative stress, and lipid synthesis [20] via regulating nuclear factor kappa-light-chain-Enhancer of activated B cells (NF-κB), nuclear factor erythroid 2-related factor 2 (Nrf2), and forkhead box protein o1 (Fox-o1) signaling pathways [16,18].

Recent studies indicate that EGCG primarily regulates lipid metabolism disorders by improving insulin sensitivity, enhancing antioxidant capacity, and inhibiting lipogenesis, whereas taurine modulates lipid metabolism by promoting fatty acid oxidation and exerting anti-inflammatory effects [3,21]. As dietary supplements, these natural bioactive compounds exhibit minimal side effects and support host gut health. A balanced gut microbiota not only facilitates nutrient absorption but also helps control blood lipid levels and fat accumulation by mitigating chronic inflammation, a key driver of lipid metabolic dysregulation [22,23]. To date, research on EGCG and taurine regulation of lipid metabolism has predominantly focused on the individual effects of single bioactive compounds. The exploitation of synergistic effects among natural products in functional food development presents a promising approach for modulating dietary patterns through targeted supplementation to optimize gut microbiota composition and subsequently ameliorate lipid metabolism disorders. This strategy offers a safe and effective non-pharmacological intervention for addressing lipid metabolic abnormalities and demonstrates significant therapeutic potential. However, the underlying mechanisms governing these synergistic interactions require further comprehensive investigation to fully elucidate their clinical applications and therapeutic efficacy.

We hypothesize that the combined administration of EGCG and taurine will demonstrate enhanced therapeutic efficacy in ameliorating lipid metabolism disorders compared to individual compound treatments, primarily through synergistic modulation of gut microbiota composition and gut–liver axis signaling pathways. Specifically, this study aims to investigate whether the combination treatment exhibits superior effects on hepatic lipid accumulation, serum lipid profiles, and intestinal barrier function while elucidating the underlying molecular mechanisms. Based on this, the present study investigated the synergistic regulatory effects of EGCG combined with taurine on lipid metabolism disorders. By elucidating the underlying mechanisms, it aims to provide novel insights for addressing lipid metabolic abnormalities and establish a theoretical foundation for developing mechanism-driven, efficacious functional food formulations targeting lipid metabolism regulation.

## 2. Materials and Methods

The human liver cancer cell line (HepG2) was purchased from the Cell Bank of the Chinese Academy of Sciences (Shanghai, China). Epigallocatechin gallate (EGCG, purity ≥ 90%) and taurine (purity ≥ 99%) were obtained from Shanghai Yuanye Bio-Technology Co., Ltd. (Shanghai, China). High-glucose Dulbecco’s Modified Eagle Medium (DMEM), fetal bovine serum (FBS), trypsin-EDTA digestion solution, and penicillin–streptomycin antibiotic solution were purchased from Thermo Fisher Scientific (Waltham, MA, USA). Kits for TG, total cholesterol (TC), HDL-C, LDL-C, MDA, GSH-Px, alkaline phosphatase (ALP), and FAS were obtained from Nanjing Jiancheng Bioengineering Institute. Modified Oil Red O Staining Kit was purchased from Shanghai Beyotime Biotechnology Co., Ltd. (Shanghai, China). Kits for free fatty acids (FFA), lipoprotein lipase (LPL), and ACC were purchased from Shanghai Enzyme-linked Biotechnology Co., Ltd. (Shanghai, China). Kits for alanine aminotransferase (ALT) and aspartate aminotransferase (AST) were purchased from Jiangsu Aidisheng Biotechnology Co., Ltd. (Yancheng, China). SOD and BCA protein quantification kits were sourced from Shanghai Sangon Biotech Co., Ltd. (Shanghai, China). Short-chain fatty acid standards (acetate, propionate, isobutyrate, butyrate, isovalerate, valerate, hexanoate) were purchased from Macklin Co., Ltd. (Shanghai, China). Chromatographic analysis was performed using a Shimadzu GC-2010 Plus gas chromatography system (Shimadzu Corporation, Kyoto, Japan). Cell lysis buffer was obtained from Shanghai Beyotime Biotechnology Co., Ltd. (Shanghai, China). β-Actin antibody and HRP-conjugated secondary antibodies were purchased from Shanghai Kangjigene Biotechnology Co., Ltd. (Shanghai, China). Antibodies against fatty acid synthase (FAS), peroxisome proliferator-activated receptor alpha (PPARα), forkhead box protein o1 (Fox-o1), and lipoprotein lipase (LPL) were purchased from Proteintech Biotechnology Co., Ltd. (Wuhan, China).

### 2.1. Cell Culture

The human liver cancer cell line (HepG2) was cultured in DMEM supplemented with 10% FBS and penicillin–streptomycin antibiotic solution under standard conditions (37 °C, 5% CO_2_, humidified atmosphere). Experimental groups were designated as normal control group (Control), oleic acid group (OA), oleic acid and EGCG group (OA + EGCG), oleic acid and taurine group (OA + Taurine), and oleic acid and EGCG with taurine synergy group (OA + EGCG + Taurine) (Table 1).

#### 2.1.1. Cell Viability Assay

When cell confluence reached approximately 90%, cells were seeded into six-well plates at a density of 2.5 mL per well in culture medium containing 10% FBS and 1% penicillin–streptomycin. Cells were further cultured until reaching 90% confluence, followed by model induction and drug treatment (OA, EGCG, and taurine were each diluted to target concentrations in complete culture medium). After treatment, cell viability was assessed using Cell Counting Kit-8 (CCK-8). Cell Counting Kit-8 was purchased from Shanghai Beyotime Biotechnology Co., Ltd. (Shanghai, China). The CCK-8 reagent was diluted 1:10 with DMEM and added to 96-well plates. Following a 30-min incubation, absorbance was measured at 450 nm using a microplate reader. Cell viability was calculated according to the following formula:

(1) $$\mathrm{Cell}\;\mathrm{viability}\left(\%\right)=\frac{{OD}_{C}\;-\;{\mathrm{OD}}_{A}}{{\mathrm{OD}}_{B}\;-\;{\mathrm{OD}}_{A}}\times 100\%,$$

OD_C_: Absorbance of sample; OD_A_: Absorbance of blank; OD_B_: Absorbance of normal cell group.

#### 2.1.2. Determination of Cellular TG Level

Preparation of Model Working Solution: To prepare the HepG2 cell lipid accumulation model working solution, 100 µL of oleic acid was added to 3.06 mL of 0.1 mol/L NaOH solution and dissolved completely with shaking in a 70 °C water bath to prepare 100 mM stock solution 1. Subsequently, 2.844 g of bovine serum albumin (BSA) was dissolved in 28.44 mL of PBS solution to prepare a 10% BSA solution, creating 10 mmol/L oleic acid stock solution 2. The two stock solutions were thoroughly mixed and filtered through a 0.22 µm organic phase filter membrane, then stored frozen at −20 °C for future use. Prior to use, the solution was incubated in a 55 °C water bath for 15 min, cooled to room temperature, and finally diluted with complete culture medium containing 1% FBS [24]. After 24 h of treatment, cells were trypsinized, collected, and centrifuged at 1000 rpm for 3 min. The supernatant was carefully removed, and the cell pellet was lysed on ice for 30–40 min using RIPA buffer containing protease inhibitor (PMSF). Triglyceride (TG) content and protein concentration in the cell lysate supernatant were determined according to the manufacturers’ instructions for the BCA protein assay kit and TG assay kit. TG content was calculated using the following formula:

(2) $$TG\left(\mathrm{mmol}/\mathrm{gprot}\right)=\frac{{OD}_{C}\;-\;{\mathrm{OD}}_{A}}{{\mathrm{OD}}_{B}\;-\;{\mathrm{OD}}_{A}}\times \;C\mathrm{standard}\;÷\;\mathrm{Cpr}$$

OD_C_: Absorbance of sample; OD_A_: Absorbance of blank; OD_B_: Absorbance of standard sample; Cstandard: Standard concentration, mmol/L; Cpr: Protein concentration of the tissue sample homogenate to be tested, gprot/L.

#### 2.1.3. Oil Red O Staining

Cells were seeded into six-well plates and treated with EGCG and taurine for 24 h. Lipid accumulation was assessed by Oil Red O staining using a commercial kit following the manufacturer’s protocol. The cell culture medium was discarded, and cells were gently rinsed once with PBS. A quantity of 1 mL of staining wash solution was added to cover the cells for 20 s, then aspirated. A quantity of 1 mL of Oil Red O working solution was added and incubated for 10–20 min, after which the staining solution was removed. A quantity of 1 mL of staining wash solution was added and allowed to stand for 30 s, then removed. Finally, cells were washed with PBS for 20 s. After staining, bright-field images were captured at 200× magnification using an inverted phase-contrast microscope. Quantitative analysis was performed using ImageJ software (2025).

### 2.2. Animals and Treatments

Specific pathogen-free (SPF) male Sprague Dawley (SD) rats (8–10 weeks old, 160–180 g) were obtained from the Experimental Animal Center of Jiangsu University (Zhenjiang, China). Animals were housed under barrier conditions (23 ± 3 °C, 40–70% humidity, 12 h light/dark cycle) with ad libitum access to food and water. After acclimatization, 60 rats were randomly assigned to six groups (n = 10 per group): normal diet—normal control (NC), High-fat emulsion—established model control (MC), simvastatin—treatment positive control (PC), EGCG treatment (EC), taurine treatment (TT), and combined EGCG and taurine treatment (ETC) (Figure S1). The NC group received daily oral gavage of saline (2 mL/100 g), while the MC, PC, EC, TT, and ETC groups were administered a high-fat emulsion (2 mL/100 g), EGCG (100 mg/kg), taurine (300 mg/kg), a combination of EGCG and taurine (100 mg/kg + 300 mg/kg), or simvastatin (10 mg/kg), respectively, for 8 consecutive weeks (simvastatin, EGCG, and taurine were all thoroughly mixed in sterile water to reach the target concentration). At the end of the experiment, all animals were euthanized by exsanguination via the carotid artery under isoflurane anesthesia (Table 2). Blood samples were collected for subsequent quantitative analysis. Fresh liver tissues were divided into portions, with some stored at −80 °C for Western blot analysis and others fixed in 4% paraformaldehyde for histopathological examination. Small intestine samples were similarly fixed in 4% paraformaldehyde for histopathological analysis. Fecal samples were stored at −80 °C for short-chain fatty acid, gut microbiota, and metabolomics analyses.

#### 2.2.1. Serum Lipids

Following blood collection, rat serum samples were separated by centrifugation at 3500 rpm for 15 min at 4 °C. Serum levels of TG, TC, HDL-C, and LDL-C were measured using commercial assay kits in strict accordance with the manufacturers’ protocols.

#### 2.2.2. Lipid Metabolism-Related Enzyme Activities and Free Fatty Acids

Serum samples collected from rats were analyzed for ALP, ACC, LPL, and FAS enzyme activities, as well as FFA levels, strictly following the instructions provided by the respective commercial assay kits.

#### 2.2.3. Liver Function Parameters

Serum levels of ALT and AST in rats were measured using commercial assay kits strictly following the manufacturers’ instructions.

#### 2.2.4. Antioxidant Enzyme Activities and Malondialdehyde Content

Serum levels of SOD, MDA, and GSH-Px were measured in rat samples using commercial assay kits according to the manufacturers’ instructions.

#### 2.2.5. Histopathological Examination

Approximately 0.1 g of liver and small intestine tissues were rinsed with physiological saline to remove blood and intestinal contents, then fixed in 4% paraformaldehyde solution. Tissue sectioning and staining were performed by Wuhan Servicebio Biotechnology Co., Ltd. (Wuhan, China). Histopathological images were captured and analyzed using an inverted microscope (Thunder Imager 3D, Wetzlar, Germany) at 200× magnification.

#### 2.2.6. Fecal Microbiota

Total genomic DNA was extracted from 200 mg of rat fecal samples using the TGuide S96 Magnetic Soil/Fecal DNA Kit. DNA quality and quantity were assessed by electrophoresis on 1.8% agarose gel, and DNA concentration and purity were determined using a NanoDrop 2000 UV-Vis spectrophotometer (Waltham, MA, USA). The hypervariable V3-V4 regions of the bacterial 16S rRNA gene were amplified using primer pair 338F (5′-ACTCCTACGGGAGGCAGCA-3′) and 806R (5′-GGACTACHVGGGTWTCTAAT-3′) [25]. Both forward and reverse 16S primers were tailed with sample-specific Illumina index sequences to enable deep sequencing analysis.

#### 2.2.7. Short-Chain Fatty Acid Determination

Samples were prepared by mixing 0.2 g of rat fecal material with 1 mL of 0.01% sulfuric acid aqueous solution. Gas chromatography analysis employed the following temperature program: initial temperature of 90 °C held for 3 min, followed by a ramp to 165 °C at 25 °C/min and maintained for 2 min, then further increased to 190 °C at 20 °C/min and held for 4 min. Carrier gas nitrogen (N_2_), hydrogen (H_2_), and air flow rates were set at 30 mL/min, 40 mL/min, and 300 mL/min, respectively. Each sample was analyzed in triplicate. Standard compounds included acetic acid, propionic acid, isobutyric acid, butyric acid, isovaleric acid, valeric acid, and hexanoic acid [26].

#### 2.2.8. Metabolomics Analysis

Fecal samples were randomly collected from three rats per group. Approximately 50 mg of each fecal sample was accurately weighed and transferred to a 2 mL microcentrifuge tube. Subsequently, 1000 μL of extraction solution containing internal standard (methanol/acetonitrile/water = 2:2:1, *v*/*v*/*v*; internal standard concentration: 20 mg/L) was added to each sample, followed by vortex mixing for 30 s to ensure homogeneous dispersion. Steel beads were then introduced into each tube, and the samples were subjected to mechanical homogenization using a ball mill grinder operating at 45 Hz for 10 min. Following homogenization, the samples underwent ultrasonic extraction for 10 min in an ice-water bath to facilitate complete metabolite extraction while preventing thermal degradation of temperature-sensitive compounds. Mobile phase compositions were identical for both positive ion mode and negative ion mode: mobile phase A consisted of 0.1% formic acid in water, while mobile phase B comprised 0.1% formic acid in acetonitrile. Metabolite detection was performed using an ultra-high-performance liquid chromatography coupled with quadrupole time-of-flight mass spectrometry (UHPLC-Q-TOF/MS) platform (Milford, MA, USA) [27]. Data acquisition was conducted on the Waters Xevo G2-XS Q-TOF system (Milford, MA, USA) operating in MS^E^ mode with dual-channel parallel acquisition. The acquisition parameters included disabled low collision energy (CE) and high collision energy ramping from 10 eV to 40 eV. Spectral acquisition rate was maintained at 0.2 s per scan. Electrospray ionization (ESI) conditions were optimized with capillary voltage set at ±2.500 V for positive and negative polarities, cone voltage at 30 V, source temperature at 100 °C, desolvation gas (N_2_) flow rate at 800 L/h with temperature at 500 °C, and nebulizer gas (N_2_) flow rate at 50 L/h [28].

#### 2.2.9. Western Blot

Total protein was extracted from 300 mg of liver tissue by homogenization in lysis buffer. Proteins were separated by sodium dodecyl sulfate-polyacrylamide gel electrophoresis (SDS-PAGE) and transferred to PVDF membranes. Membranes were blocked with 5% non-fat dry milk for 1 h at room temperature, followed by overnight incubation at 4 °C with primary antibodies against PPARα, FAS, LPL, Fox-o1, and β-actin. After three washes with TBST buffer, membranes were incubated with HRP-conjugated secondary antibodies for 1 h at room temperature. Chemiluminescent signals were captured using a Tanon 4800 multi-CCD imaging system, and protein band intensity was quantified using ImageJ image analysis software.

#### 2.2.10. Statistical Analysis

Experimental data were presented as mean ± standard error of the mean (SEM) and analyzed using one-way analysis of variance (ANOVA) with GraphPad Prism 9 software (GraphPad Software, USA) (San Diego, CA, USA). Statistical significance was determined at *p* < 0.05. Gut microbiota analysis was conducted using operational taxonomic unit (OTU) clustering analysis according to established protocols [21], with data visualization performed on the BMKCloud platform (http://www.biocloud.net (accessed on 25 March 2025)). Raw data acquired using MassLynx V4.2 underwent peak extraction and peak alignment through Progenesis QI (2020.6) software. Metabolite identification was performed using the online METLIN database, public databases, and proprietary BMK databases integrated within Progenesis QI software, with simultaneous theoretical fragment identification for compound confirmation.

## 3. Results and Analysis

### 3.1. Effects of EGCG and Taurine on Cellular Viability

**Figure 1:**
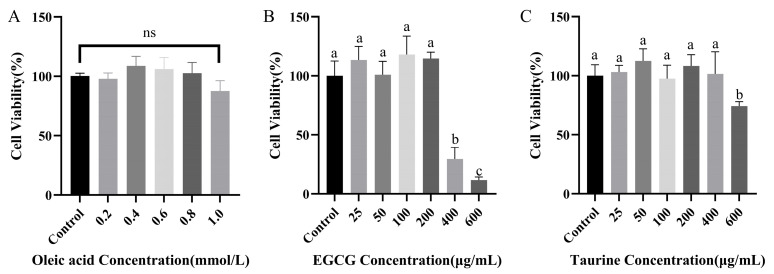
Cell viability assay. (**A**) Effects of different concentrations of oleic acid on cell viability. (**B**) Effects of different concentrations of EGCG on cell viability. (**C**) Effects of different concentrations of taurine on cell viability. a–c: Different lowercase letters indicate statistically significant differences (*p* < 0.05). Data are means ± standard error of the mean (SEM) (n = 3). ns: statistically non-significant results (*p* > 0.05).

Cell viability is an important indicator for assessing the proportion of living cells within a cell population and can be used to evaluate experimental conditions. As shown in Figure 1, oleic acid (OA) at concentrations ranging from 0 to 1.0 mmol/L had no significant effect on cell viability (*p* > 0.05). Although treatment with 0–200 μg/mL EGCG caused some fluctuations in HepG2 cell survival rates, there was no significant difference compared to the control group (*p* > 0.05). However, at 400 μg/mL, cell viability decreased to approximately 40%, indicating cytotoxicity at this concentration. Taurine at concentrations of 0–400 μg/mL did not significantly affect HepG2 viability (*p* > 0.05 vs. control group), whereas at 600 μg/mL, cell viability dropped below 80%, indicating a toxic effect. Therefore, subsequent experiments used EGCG concentrations below 400 μg/mL and EGCG and taurine concentrations below 600 μg/mL.

### 3.2. Effects of EGCG and Taurine on Triglyceride Levels in HepG2 Cells

**Figure 2:**
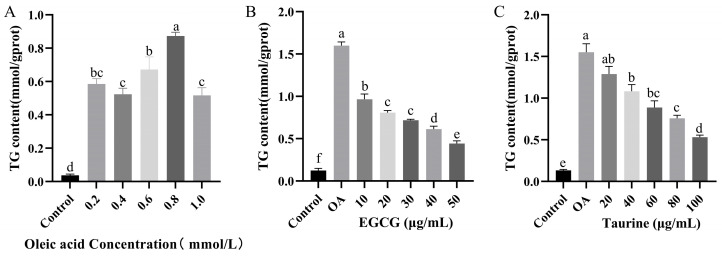

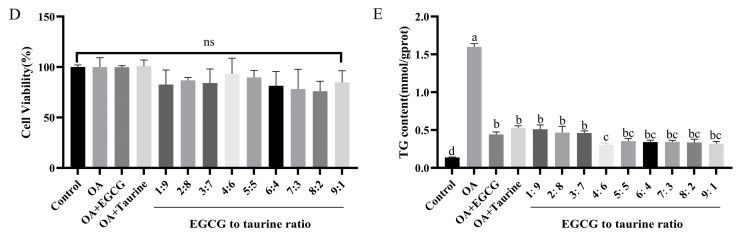
Triglyceride content. (**A**) Effects of different concentrations of oleic acid on triglyceride content. (**B**) Effects of different concentrations of EGCG on triglyceride content. (**C**) Effects of different concentrations of taurine on triglyceride content. (**D**) Effects of varying EGCG-to-taurine ratios on cellular viability. (**E**) Effects of varying EGCG-to-taurine ratios on triglyceride content. a–f: Different lowercase letters indicate statistically significant differences (*p* < 0.05). Data are means ± SEM (n = 3). ns: statistically non-significant results (*p* > 0.05).

TG levels are key biomarkers for assessing lipid metabolism disorders, with elevated TG closely associated with dysregulated lipid metabolism. Experimental data showed that cellular TG levels peaked at an oleic acid concentration of 0.8 mmol/L. The most significant inhibition of TG production in HepG2 cells was observed at 50 μg/mL EGCG and 100 μg/mL taurine (*p* < 0.05). Compared to the OA group, TG content in groups OA + EGCG and OA + Taurine decreased by 72.4% and 65.8%, respectively. Further analysis revealed that a combined treatment of EGCG and taurine at a volume ratio of 4:6 (mass ratio 1:3) resulted in the lowest intracellular TG content, representing reductions of 27.8% and 39.6% compared with OA + EGCG and OA + Taurine, respectively (*p* < 0.05). These results indicate a significant synergistic lipid-lowering effect at an EGCG to taurine mass ratio of 1:3, without cytotoxicity (Figure 2).

### 3.3. Oil Red O Staining Analysis

**Figure 3:**
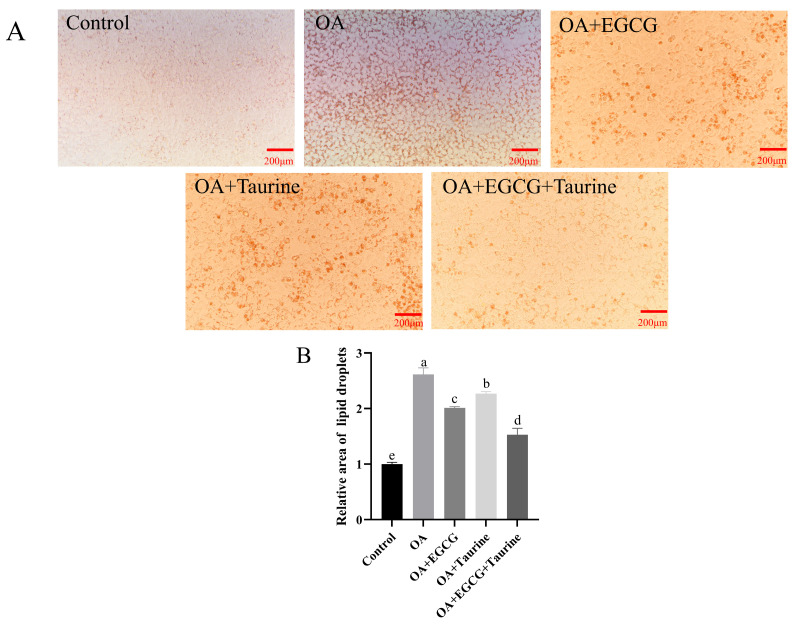
Oil Red O staining results. (**A**) Oil Red O staining; (**B**) quantitative analysis of Oil Red O Staining. a–e: Different lowercase letters indicate statistically significant differences (*p* < 0.05). Data are means ± SEM (n = 3).

Oil Red O is a lipophilic dye that binds to fatty acids or lipid droplets, staining adipose tissue or cells red or orange-red. As shown in Figure 3, compared to the OA, group OA + EGCG, OA + Taurine, and OA + EGCG + Taurine all exhibited significant reductions in intracellular triglyceride accumulation. Notably, the lipid droplet area of OA + EGCG + Taurine decreased by 42.3% and 38.7% compared to OA + EGCG and OA + Taurine, respectively (*p* < 0.05), representing the most pronounced reduction (Figure 1C). These results visually confirm the synergistic lipid-lowering effect of EGCG and taurine.

### 3.4. Effects of EGCG and Taurine on the Body Weight of Rats Subjected to Long-Term HFD

EGCG, taurine, and their combined intervention significantly affected body weight changes in rats fed a long-term high-fat diet (HFD). On the 56th day of the experiment, the body weight in MC increased by 27% compared to NC (*p* < 0.05). In contrast, EC, TT and ETC showed body weight reductions of 8.83% (*p* < 0.05), 5.47% (*p* < 0.05), and 18.5% (*p* < 0.05), respectively, relative to the MC group (Figure S2). The weight loss effect in the ETC group was significantly greater than that in EC and TT (*p* < 0.05), indicating a synergistic effect of EGCG and taurine on weight reduction. The PC group, treated with simvastatin—which exerts multifaceted regulation on lipid metabolism, muscle function, and appetite—showed the greatest weight reduction, with a 24.3% decrease compared to the MC group [29] (*p* < 0.05).

### 3.5. Analysis of the Effects of EGCG and Taurine on Rat Blood Lipids, Lipid Synthesis and Metabolism-Related Enzymes, Key Hepatic Function Enzymes, and Antioxidant Enzymes

#### 3.5.1. Effects of EGCG and Taurine on Blood Lipid Levels in Rats

After 56 days of HFD feeding, the rats exhibited characteristic dyslipidemia, with significant increases in TG, TC, and LDL-C levels, accompanied by a decrease in HDL-C (Table 1). Compared to NC, MC showed 3.16-fold, 13.27-fold, and 29.1-fold increases in TC, TG, and LDL-C, respectively, and a 1.03-fold decrease in HDL-C (*p* < 0.05). Oral administration of EGCG, taurine, and their combination significantly reduced serum TC, TG, and LDL-C levels and markedly increased HDL-C levels compared to the MC group (*p* < 0.05). Specifically, TG levels in the ETC group decreased by 23.07% and 38.48% compared to the EC and TT groups, respectively (*p* < 0.05). TC levels in the ETC group were reduced by 33.3% and 41.8%, and LDL-C levels decreased by 32.3% and 29.2% relative to the EC and TT groups, respectively. Furthermore, HDL-C levels in the ETC group increased by 12.7% and 33.5% compared to EC and TT, respectively. These results indicate that EGCG, taurine, and their combination significantly lower serum TG, TC, and LDL-C levels while elevating HDL-C in rats fed HFD, with the combination exerting a more effective synergistic effect in regulating lipid profiles.

#### 3.5.2. Effects of EGCG and Taurine on Lipid Metabolism-Related Enzyme Activities and FFA Levels in Rats

The activities of ALP, ACC, FAS, LPL, and the levels of FFA were measured to investigate the effects of EGCG and taurine on lipid metabolism-related enzymes and FFA levels (Table 1). Under conditions of lipid metabolism disorder induced by long-term HFD, ALP, ACC, FAS, and FFA levels in rat serum typically increase, while LPL levels decrease. Compared to NC, group MC showed significant increases in ALP, ACC, FAS, and FFA levels by 3.06-, 1.35-, 1.85-, and 1.77-fold, respectively, and a 58.62-fold decrease in LPL activity (*p* < 0.05). Following oral administration of EGCG, taurine, and their combination, the ALP levels in the EC, TT, and ETC groups decreased significantly by 30.72%, 20.94%, and 51.05%, respectively, compared to MC (*p* < 0.05). FAS levels decreased by 22.39%, 23.94%, and 35.41% (*p* < 0.05), while LPL activity increased by 21.14-, 7.38-, and 31.31-fold (*p* < 0.05). FFA levels were reduced by 33.94%, 29.86%, and 42.28% (*p* < 0.05). A significant reduction in ACC levels was observed only in the ETC group, with a 35.66% decrease compared to MC (*p* < 0.05). These findings indicate that EGCG and taurine exert significant modulatory effects on lipid synthesis enzyme activities. Notably, compared to EC and TT, ETC showed further reductions in ALP levels by 29.41% and 38.09%, respectively; no significant difference in ACC levels (*p* > 0.05); decreases in FAS levels by 16.76% and 15.07%; increases in LPL activity by 52.9% and 2.85-fold; and reductions in FFA levels by 12.93% and 18%. These results demonstrate a synergistic enhancement by EGCG and taurine on the activities of lipid metabolism-related enzymes ALP, FAS, LPL, and FFA content in rats with lipid metabolic disorders, suggesting that their combination may more effectively improve liver function, promote hydrolysis of circulating triglycerides, and reduce lipid accumulation.

#### 3.5.3. Effects of EGCG and Taurine on Liver Function-Related Enzyme Activities in Rats

The impact of EGCG and taurine on liver function in HFD rats was assessed by measuring serum ALT and AST levels. As shown in Table 3, compared to NC, MC exhibited significant increases in ALT and AST levels by 1.51- and 3.74-fold, respectively (*p* < 0.05). Both ALT and AST levels were significantly reduced following intervention with EGCG and taurine (*p* < 0.05). Notably, the ETC group showed significantly lower ALT and AST levels compared to EC and TT (*p* < 0.05), with ALT decreased by 50.88% and 54.48%, and AST decreased by 18.2% and 23.43%, respectively. These findings indicate that the combination of EGCG and taurine provides superior protection against liver damage induced by lipid accumulation and degeneration compared to individual treatments. Importantly, ALT and AST levels in the ETC group were also lower than those in the PC group, suggesting that the synergistic effect of EGCG and taurine offers better hepatoprotective benefits and mitigates liver injury more effectively than simvastatin.

#### 3.5.4. Effects of EGCG and Taurine on Antioxidant Enzyme Activities and MDA Levels in Rats

SOD, GSH-Px, and MDA play crucial roles in lipid metabolism regulation, and their activity changes reflect oxidative stress induced by lipid metabolic disorders. Long-term HFD feeding caused significant alterations in serum SOD and GSH-Px activities and MDA content in the MC group compared to the NC group (*p* < 0.05). Specifically, SOD and GSH-Px activities decreased by 51.48% and 47.74%, respectively, while MDA content increased 1.73-fold, indicating disruption of the oxidative balance and oxidative stress damage induced by HFD. Treatment with EGCG and taurine significantly increased serum SOD and GSH-Px activities and reduced MDA levels, indicating attenuated oxidative damage in rats. No significant difference was found in the SOD activity among different groups (*p* > 0.05); however, the levels of MDA decreased by 51.17% and 48.22% relative to EC and TT, respectively, while GSH-Px activity increased by 45.58% and 10.88%. These results demonstrate that EGCG and taurine synergistically enhance antioxidant capacity, thereby significantly mitigating oxidative stress damage induced by HFD.

### 3.6. H&E Staining of Liver and Small Intestine Tissues

**Figure 4:**
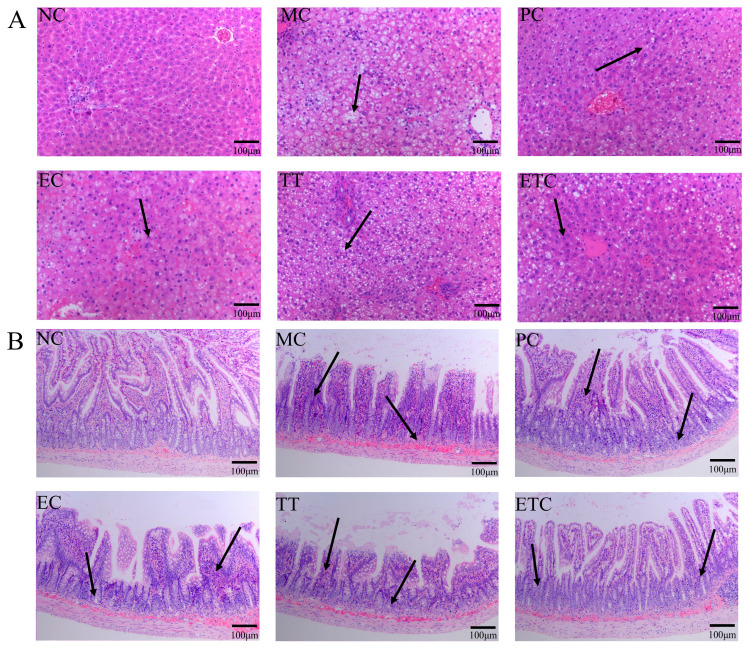
Effects of EGCG and taurine treatment on liver and small intestine histopathology in HFD rats. (**A**) H&E staining results of the liver; (**B**) H&E staining results of the small intestine. NC: normal diet control, MC: high-fat emulsion established model control, PC: simvastatin treatment positive control, EC: EGCG treatment, TT: taurine treatment, ETC: combined EGCG and taurine treatment. Black arrows indicate nuclear changes and inflammatory infiltration.

Long-term HFD induces liver injury and disrupts the integrity of the intestinal barrier, leading to gut microbiota dysbiosis, which further exacerbates liver damage and increases intestinal mucosal permeability [30]. As shown in Figure 4, the MC group exhibited pronounced hepatic steatosis characterized by obvious lipid vacuoles, disorganized cellular arrangement, and evident nuclear deformation in liver cells. The small intestine also displayed significant pathological changes, including villus atrophy, mucosal thinning, and severe inflammatory infiltration. EGCG and taurine administered individually moderately ameliorated HFD-induced liver and intestinal tissue damage, though the effects were limited. Combined treatment with EGCG and taurine demonstrated a synergistic effect, producing the most significant improvement in pathological conditions of both liver and small intestine tissues, with the protective effect on the small intestine reaching levels close to those of the normal control group.

### 3.7. Fecal Microbiota Analysis

Lipid metabolism disorders can exacerbate gut microbiota dysbiosis, which in turn further aggravates metabolic dysfunction [21]. Long-term HFD feeding significantly reduced microbial abundance; compared to the NC, the MC showed marked decreases in Chao1, Ace, Shannon, and Simpson indices by 42.1%, 38.7%, 29.3%, and 33.5%, respectively (*p* < 0.05), indicating reduced richness and diversity of gut microbiota under prolonged HFD conditions. Treatment with EGCG and taurine elevated all four indices, suggesting their potential to restore microbial abundance and diversity in rats. Notably, the ETC exhibited significantly higher Ace and Simpson indices than the EC and TT groups (*p* < 0.05), demonstrating a synergistic effect in enhancing gut microbiota richness and diversity in HFD-fed rats (Table 4).

Principal component analysis (PCA) revealed significant clustering differences among groups (*p* < 0.05), with clear separation between NC and MC, indicating that HFD substantially altered microbiota composition. Spatial distribution in the ETC group further suggested that combined EGCG and taurine intervention has superior modulatory effects on microbial community structure compared to monotherapies. Flower diagram analysis identified 109 core OTUs shared among groups; the MC group contained 453 unique OTUs, whereas the ETC group harbored 827 unique OTUs, comparable to the PC group and significantly exceeding those in the EC (656) and TT (681) groups, implying that EGCG and taurine synergy may enrich distinctive microbial taxa to achieve enhanced regulation (Figure S3).

**Figure 5:**
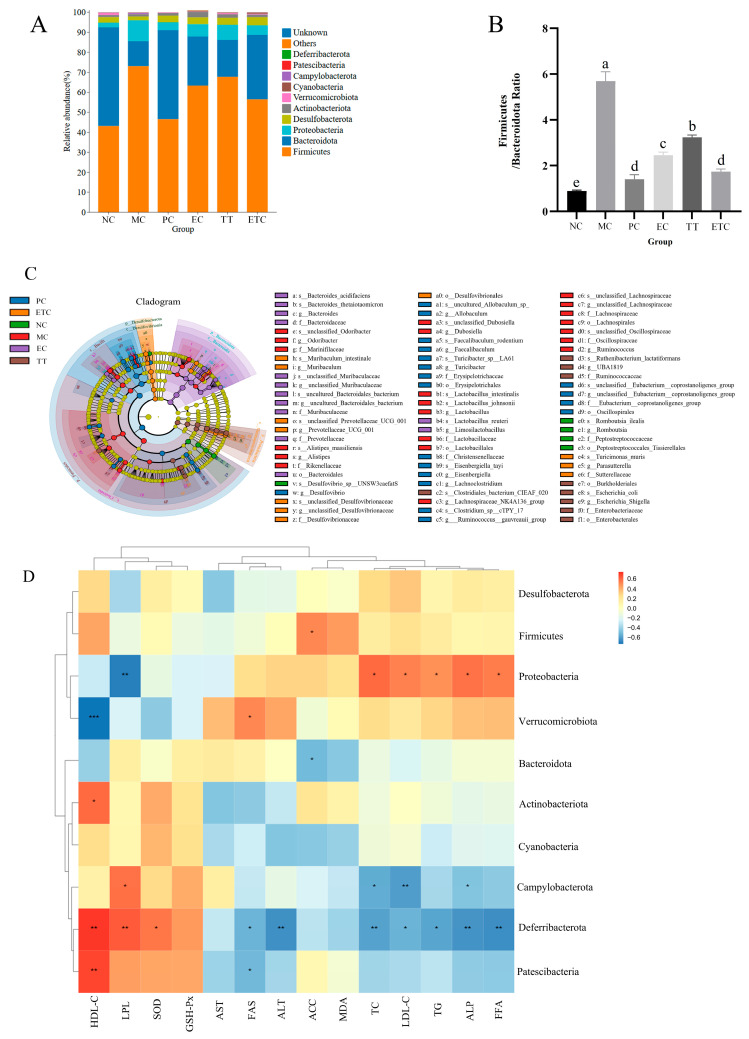
Effects of EGCG and taurine on rat gut microbiota. (**A**) Distribution of gut microbiota at the phylum level; (**B**) Firmicutes-to-Bacteroidota ratio; (**C**) Phylogenetic tree from phylum to genus levels; (**D**) Heatmap of correlations between gut microbiota and serum biomarkers. NC: normal diet control, MC: high-fat emulsion established model control, PC: simvastatin treatment positive control, EC: EGCG treatment, TT: taurine treatment, ETC: combined EGCG and taurine treatment. Data are means ± SEM (n = 3). a–e: Different letters in the same column indicate significant differences in the numerical values (*p* < 0.05). * denotes *p* < 0.05, ** denotes *p* < 0.01, *** denotes *p* < 0.001.

Phylum level analysis showed that HFD induced an abnormal increase in the proportion of *Bacteroidota* and a significant decrease in *Firmicutes* in rat feces, constituting a classic pattern of lipid metabolic imbalance. Additionally, *Proteobacteria* abundance markedly increased. The combined intervention reduced the *Firmicutes*/*Bacteroidota* (F/B) ratio by 63.4% compared to the MC group (Figure 5A,B). Phylogenetic tree analysis (Figure 5C) demonstrated significant taxonomic differences in MC group gut microbiota, characterized by enrichment of *Firmicutes* taxa, particularly *Lachnospiraceae*, *Ruminococcaceae*, and *Clostridiaceae*. In contrast, the NC group exhibited a more balanced community dominated by *Bacteroidota* families such as *Bacteroidaceae* and *Prevotellaceae*. The EC group showed moderate enrichment of *Bacteroidota* lineages and a significant increase in *Akkermansia muciniphila*. While EC promoted proliferation of *Lactobacillaceae* members, it did not fully restore microbial structure. The TT group displayed enrichment of *Proteobacteria*, especially *Desulfovibrionaceae*, but *Firmicutes* remained predominant. Additionally, TT increased specific *Clostridiales* absent in other groups, indicating taurine’s targeted therapeutic effects without full microbiota normalization. The phylogenetic profile of ETC was more similar to NC than EC or TT, exhibiting comparable distributions across multiple taxonomic levels. Importantly, ETC restored key *Bacteroidota* taxa while significantly reducing HFD-induced overgrowth of specific *Firmicutes* clades.

Correlation analysis (Figure 5D) showed positive correlations of ACC with *Firmicutes* and negative correlations with *Bacteroidota*. *Proteobacteria* correlated positively with TC, LDL-C, TG, ALP, and FFA, but negatively with LPL. *Deferribacterota* correlated positively with HDL-C, LPL, and SOD, and negatively with FAS, ALT, TC, LDL-C, TG, ALP, and FFA. These results indicate the interactions between serum biochemical parameters and gut microbiota, suggesting that EGCG and taurine exert beneficial regulatory effects on the gut microbiome in lipid metabolism disorders.

### 3.8. Short-Chain Fatty Acid Analysis

Short-chain fatty acids (SCFAs), as key microbial metabolites, mediate host–microbiome interactions and significantly influence host metabolic health [31]. In this study, systematic analysis of seven major SCFAs in rat fecal samples revealed that HFD induced significant alterations in SCFA levels, whereas combined intervention with EGCG and taurine demonstrated a pronounced restorative effect. Compared to the NC group, primary SCFAs in the MC group were significantly reduced: acetate decreased by 49.4%, propionate by 48.4%, and butyrate by 28.0%. The ETC group restored acetate levels to 77.7% of those in the NC group, significantly outperforming either EGCG or taurine alone. A similar trend was observed for propionate, with the combined intervention achieving a 90.4% restoration. Among branched-chain fatty acids, isobutyrate and isovalerate exhibited parallel responses: HFD decreased isobutyrate by 45.5% and isovalerate by 41.2%. ETC treatment restored these to 90.8% and 87.7% of NC levels, respectively, suggesting that it is markedly superior to single interventions. Additionally, valerate and hexanoate levels were reduced by 27.2% and 32.4% due to HFD, with EGCG and taurine combined treatment restoring them to 87.5% and 88.0% of NC values, respectively (Table 5). These findings highlighted the efficacious role of EGCG and taurine synergy in restoring SCFA profiles disrupted by high-fat diet feeding.

### 3.9. Metabolomics Result

To evaluate intergroup differences in the metabolic profiles of rats, untargeted metabolomics analysis was performed. Principal Coordinates Analysis (PCoA) revealed clear separation in metabolic distribution among groups. Orthogonal Partial Least Squares Discriminant Analysis (OPLS-DA) showed distinct clustering, with the ellipses representing the 95% confidence intervals. All groups were significantly separated, indicating pronounced metabolic differences. Permutation tests (Figure S4A–H) demonstrated positive slopes in the Q2Y regression lines, with all Q2Y values exceeding 0.9, confirming the reliability of the OPLS-DA models. Venn diagram analysis further identified 46 and 567 differential abundance metabolites between the MC and ETC/NC groups, respectively, indicating that HFD induced significant metabolic disturbances, while ETC intervention effectively reshaped the metabolic profile (Figure S5A,B) [25].

**Figure 6:**
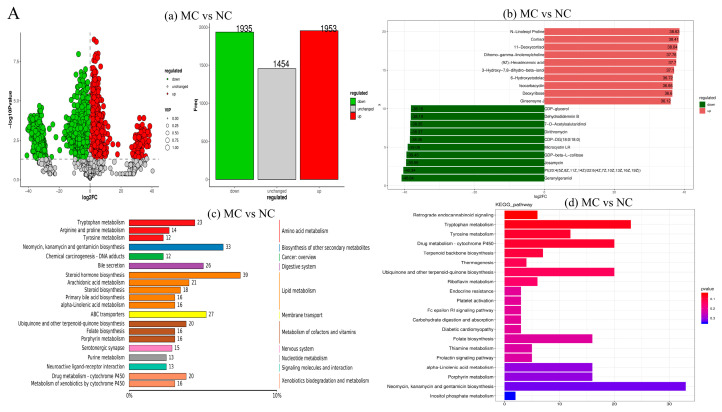

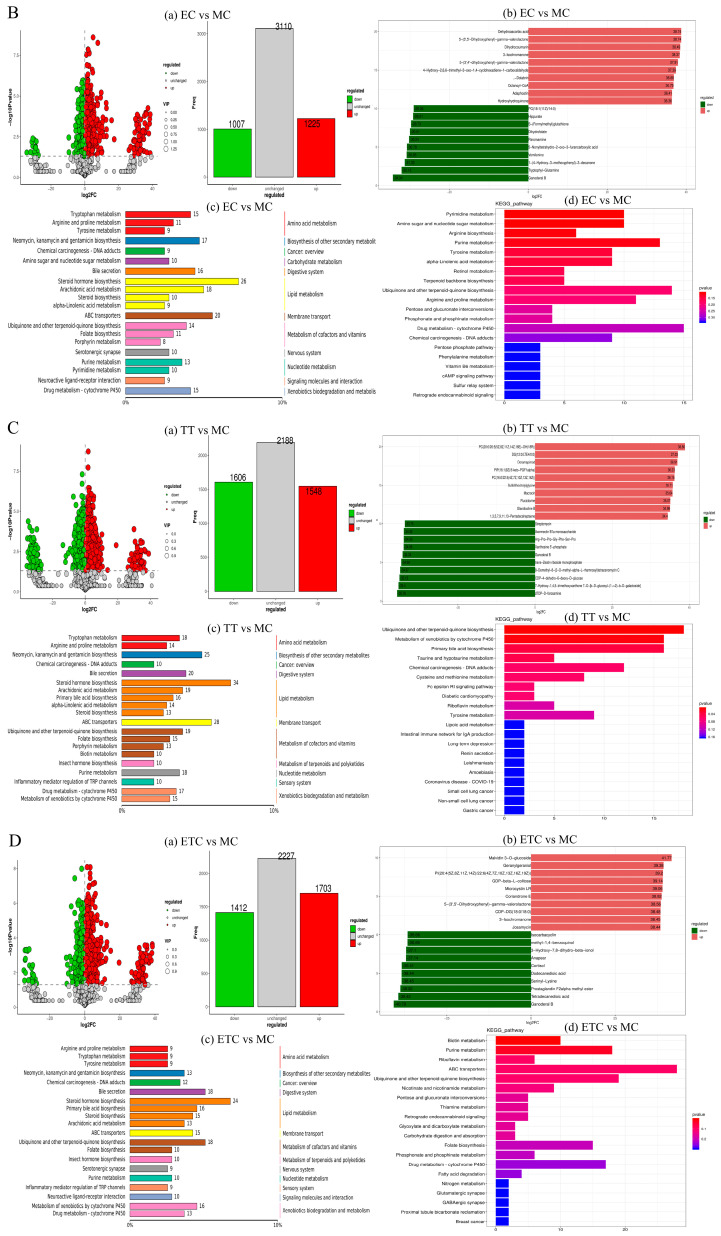

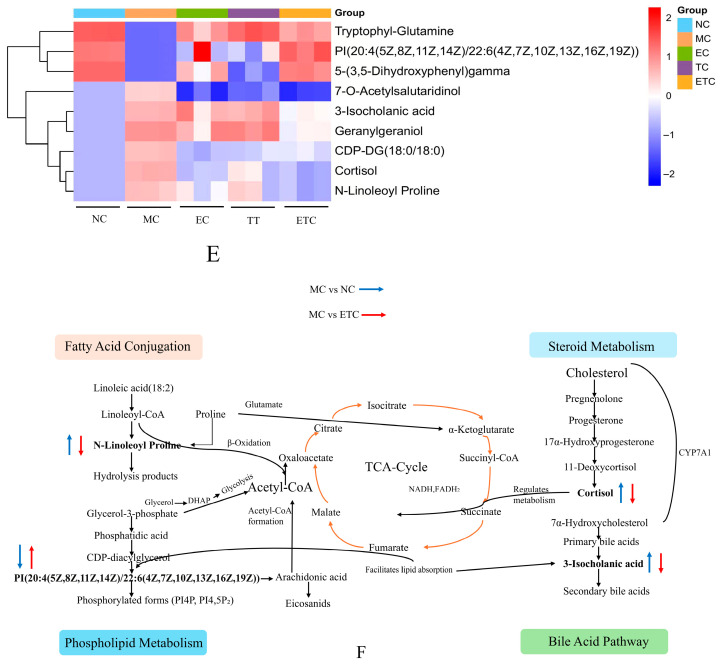
Metabolomic analysis. (**A**) MC vs. NC; (**B**) EC vs. MC; (**C**) TT vs. MC; (**D**) ETC vs. MC; (**E**) cluster heatmap of metabolites among groups; (**F**): synergistic modulation of metabolic pathways by EGCG and taurine in HFD-fed mice. (**a**): Volcano plot; (**b**): differential fold-change bar chart; (**c**): classification of differential metabolite pathways; (**d**): KEGG pathway bar chart. NC: normal diet control, MC: high-fat emulsion established model control, EC: EGCG treatment, TT: taurine treatment, ETC: combined EGCG and taurine treatment.

Figure 4 illustrates the metabolic alterations induced by HFD and interventions with EGCG and taurine (statistical thresholds: *p* < 0.05, VIP > 1). Under prolonged HFD conditions, upregulated metabolites were predominantly lipids, such as N-linoleoyl proline and cortisol, indicating lipid metabolic disorder in rats [32]. Downregulated metabolites were mainly carbohydrate derivatives, including GDP-glycerol, reflecting disruptions in energy metabolism pathways (Figure 6A). Following EGCG monotherapy, upregulated metabolites primarily comprised polyphenolic compounds and dehydroascorbic acid, while downregulated metabolites were mainly glutathione metabolism intermediates and dihydrofolate, suggesting oxidative stress challenges with a compensatory antioxidant response induced by EGCG (Figure 6B) [33]. Taurine monotherapy led to upregulation of phospholipids and flunisolide, reflecting taurine’s anti-inflammatory properties and its regulatory effect on phospholipid metabolism in HFD conditions [34]. Downregulated metabolites included antibiotics such as streptomycin and nucleotide sugar metabolism intermediates, indicating reduced microbial defense demands and more rationalized energy allocation under taurine treatment (Figure 6C) [35]. Combined EGCG and taurine treatment upregulated polyphenols, ubiquinones/terpenoids, indicating amelioration of lipid peroxidation and inflammation. Downregulated metabolites were mainly fatty acids, such as tetracosanoic acid, demonstrating significant suppression of fatty acid synthesis induced by HFD [36] (Figure 6D). It was identified that lipid and amino acid metabolism are the key disrupted pathways under HFD-induced lipid metabolic disorders, alongside impacts on coenzyme, nucleotide metabolism, membrane transport, and signal transduction systems (Figure 6A) [37,38,39]. EGCG monotherapy partially improved lipid and amino acid metabolism relative to the MC group (Figure 6B), while taurine monotherapy enhanced detoxification, drug metabolism, and energy/coenzyme pathways (Figure 6C). The combined intervention synergistically balanced metabolic states, integrating EGCG’s potent antioxidant and taurine’s anti-inflammatory effects. Steroid hormone biosynthesis and arachidonic acid metabolism were moderately regulated, whereas steroid biosynthesis and α-linolenic acid metabolism decreased, indicating alleviated lipid metabolic burden [40]. ABC transporters involved in membrane transport maintained moderate activity. Secondary metabolite biosynthesis pathways for neomycin, kanamycin, and gentamicin, along with bile secretion in the digestive system, were at intermediate levels. Aminosugar and nucleotide sugar metabolism pathways in carbohydrate metabolism suggested diversified energy metabolism (Figure 6D) [41]. KEGG (Kyoto Encyclopedia of Genes and Genomes) enrichment analysis revealed that the MC group exhibited the most imbalanced and aberrant metabolic profile, with abnormally enriched biosynthesis of antibiotics such as neomycin surpassing all other pathways, reflecting severe metabolic disorder. The activation of the diabetic cardiomyopathy pathway indicated that metabolic dysfunction affected cardiac function in MC rats (Figure 6A) [42]. EGCG monotherapy maintained a functional albeit stressed metabolic state, with significant yet non-extreme enrichment of purine and pyrimidine metabolism pathways supporting normal nucleic acid synthesis and coordinated amino acid metabolism (Figure 6B). Taurine monotherapy showed marked upregulation of ubiquinone biosynthesis and cytochrome P450 xenobiotic metabolism, indicative of pronounced oxidative and toxic stress [43]. Heightened primary bile acid biosynthesis suggested increased hepatic metabolic burden [44]. Multiple disease-related pathways were also enriched, reflecting multisystem involvement (Figure 6C). The combined EGCG and taurine treatment exhibited a more balanced and efficient metabolic profile compared to monotherapies, characterized by significant enrichment of ABC transporters and coordinated activation of vitamin metabolism pathways including biotin, riboflavin, thiamine, and folate. This supports diverse coenzyme functions, efficient substance transport, moderate detoxification capacity, and balanced energy metabolism in the ETC group (Figure 6D) [45,46].

Cluster heatmap analysis revealed an inverse metabolic profile between the MC and the NC groups. Metabolites such as tryptophanyl-glutamine (dipeptide), phosphatidylinositol PI (20:4/22:6) (phospholipid), and 5-(3,5-dihydroxyphenyl)-γ-compound (phenolic compound) were elevated in the NC group, whereas MC rats exhibited significantly increased levels of 7-O-acetylstachydrine (alkaloid), 3-isocholanate (bile acid), geranyl geraniol (isoprenoid), CDP-DG (18:0/18:0) (phospholipid precursor), cortisol (glucocorticoid), and N-linoleoyl proline (N-acyl amino acid). These alterations indicate widespread disruptions in amino acid, lipid, and steroid metabolic pathways, consistent with characteristic metabolic dysregulation. The ETC group demonstrated significantly better recovery of alkaloids (7-O-acetylstachydrine), phospholipid precursors (CDP-DG (18:0/18:0)), and N-acyl amino acids (N-linoleoyl proline) compared to the EC group, indicating a synergistic and complementary effect on metabolic pathways. Compared to the TT group, group ETC showed superior restoration of phospholipids (PI (20:4/22:6)), isoprenoids (geranyl geraniol), and phenolic compounds (5-(3,5-dihydroxyphenyl)-γ-compound), suggesting that combined intervention can concurrently regulate multiple dysregulated pathways. Although monotherapies partially ameliorated metabolic disturbances, the EGCG–taurine combination achieved more comprehensive metabolic normalization by targeting multiple biochemical pathways impaired by high-fat diet feeding (Figure 6E). The metabolic pathway diagram is presented in Figure 6F.

### 3.10. EGCG and Taurine Regulate Glucose and Lipid Metabolism via PPARα/FAS Axis in Fatty Acid Synthesis/Catabolism

**Figure 7:**
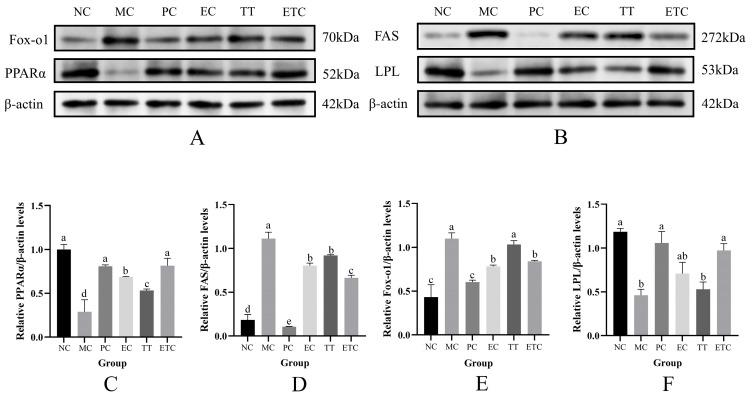
Effects of EGCG and taurine on the expression of proteins related to fatty acid synthesis and catabolism. (**A**) Fox-o1 and PPARα protein bands; (**B**) FAS and LPL protein bands. (**C**) Relative expression level of PPARα; (**D**) relative expression level of FAS; (**E**) relative expression level of Fox-o1; (**F**) relative expression level of LPL; NC: normal diet control, MC: high-fat emulsion established model control, PC: simvastatin treatment positive control, EC: EGCG treatment, TT: taurine treatment, ETC: combined EGCG and taurine treatment. Data are means ± SEM (n = 2). a–e: Different letters in the same column indicate significant differences in the numerical values (*p* < 0.05).

Imbalance in the PPARα/Fox-o1 signaling pathway can induce dysregulation of FAS and LPL, a mechanism closely associated with visceral obesity and hypertriglyceridemia [47]. PPARα and Fox-o1 act as antagonistic regulators of lipid metabolism: activation of PPARα enhances lipolysis by upregulating LPL and suppressing FAS expression, whereas Fox-o1 activation promotes FAS expression and inhibits LPL activity, thereby driving lipogenesis [48]. As shown in Figure 7, compared to the NC group, the MC group exhibited significantly increased hepatic expression of FAS and Fox-o1 and decreased expression of PPARα and LPL (*p* < 0.05), indicating impaired lipid catabolism and enhanced lipogenesis in the liver under long-term high-fat diet conditions, leading to hepatic lipid accumulation. Following treatment with EGCG, taurine, and their combination, protein expression levels of FAS and Fox-o1 were significantly downregulated, while PPARα and LPL levels were markedly upregulated in the model rats compared to MC (*p* < 0.05). This suggests that EGCG and taurine improve hepatic lipid catabolism and suppress fatty acid synthesis. Furthermore, combined treatment with EGCG and taurine significantly increased hepatic PPARα expression and decreased FAS protein levels compared to monotherapies (*p* < 0.05), indicating superior enhancement of lipid breakdown and inhibition of fatty acid synthesis in the liver. Notably, although no significant differences were observed between the combined and EGCG-alone groups in Fox-o1 and LPL expression (*p* > 0.05), the combined treatment markedly reduced these proteins compared to taurine alone (*p* < 0.05). This suggests that taurine alone is less effective than EGCG or their combination in promoting lipolysis and inhibiting lipogenesis and may exert its effects via alternative pathways.

## 4. Discussion

Lipid metabolism disorders are primarily induced by poor lifestyle habits or genetic factors [49]. Associated complications include obesity, fatty liver disease, cardiovascular disease, gut microbiota dysbiosis, and metabolic dysfunction [50]. Maintaining normal body weight and regulating dyslipidemia represent the principal approaches for preventing and treating lipid metabolism disorders [51]. EGCG and taurine have been demonstrated to possess therapeutic potential for lipid metabolism disorders [3,52]. Furthermore, modulation of gut microbiota, improvement of metabolic imbalance, and intervention in lipid synthesis/catabolism signaling pathways may provide breakthrough strategies for managing lipid metabolism disorders and their complications [53,54]. This study established an HepG2 cell lipid accumulation model to determine the optimal mass ratio of EGCG to taurine (1:3), which significantly reduced triglyceride levels compared to individual treatments. Subsequently, we systematically investigated the synergistic mechanism of EGCG and taurine in regulating lipid metabolism disorders in rats through comprehensive analysis of body weight, serum lipid levels, lipid metabolism-related enzyme activities, liver function, antioxidant enzyme activities, gut microbiota, short-chain fatty acids, metabolomics, and the PPARα/FAS signaling pathway, providing scientific evidence for evaluating polyphenol–taurine complexes in improving lipid metabolism disorders.

Long-term HFD induces hepatic steatosis through disruption of lipid homeostasis [55]. Our study demonstrates that combined EGCG and taurine treatment exhibits superior efficacy compared to monotherapy in ameliorating diet-induced metabolic dysfunction. Significant improvements in lipid profiles, including marked reductions in TC, TG, and LDL-C alongside elevated HDL-C, align with previous findings on the synergistic effects of tea polyphenols and taurine in nonalcoholic steatohepatitis treatment [56]. The combination intervention demonstrated superior regulatory effects on lipogenic and lipolytic enzyme activities compared to individual treatments. Specifically, the EGCG–taurine combination significantly inhibited key lipogenic enzymes including ACC and FAS, while simultaneously enhancing LPL activity and reducing FFA levels. Hepatoprotective effects were evidenced by decreased ALT/AST levels and enhanced antioxidant capacity, characterized by increased SOD and GSH-Px activities alongside reduced MDA levels. These findings indicate that the combination therapy may preserve hepatocyte integrity through synergistic antioxidant mechanisms.

Gut microbiota dysbiosis represents a critical factor influencing lipid metabolism and intestinal barrier function, playing a pivotal role in metabolic diseases [57]. This study demonstrates that EGCG and taurine synergistically regulate gut microbial community structure, significantly ameliorating lipid metabolism abnormalities. The *Firmicutes*/*Bacteroidetes* ratio (F/B) serves as a key biomarker of metabolic health, with elevated ratios closely associated with metabolic dysfunction [58]. While EGCG monotherapy enhances beneficial *Akkermansia* abundance and taurine selectively enriches *Clostridiales* members, their combined intervention comprehensively reshapes microbial composition and significantly reduces the F/B ratio. Recent evidence indicates that EGCG preferentially enriches *Akkermansia* and modulates circulating fatty acid profiles, whereas taurine supplementation reduces *Proteobacteria* abundance and promotes SCFA production [59,60]. The observed negative correlation between total cholesterol and *Bacteroides* abundance aligns with recent metabolomic analyses demonstrating that tea polyphenols reshape gut microbiota to improve lipid metabolism [21]. H&E revealed that EGCG–taurine combination therapy effectively preserves intestinal villus architecture and prevents inflammatory infiltration, advantages not observed in monotherapy groups. This intestinal barrier protection mechanism likely stems from synergistic enhancement of microbial stability and promotion of SCFA synthesis, particularly butyrate production by *Lachnospiraceae* and related taxa. Furthermore, the combination may strengthen antioxidant defenses through complementary pathways and attenuate oxidative stress-mediated intestinal epithelial cell apoptosis. These mechanisms suggest that EGCG and taurine improve metabolic dysfunction by synergistically modulating gut microbiota and enhancing intestinal barrier integrity, targeting the gut–liver axis.

Metabolomics analysis comprehensively elucidated the global metabolic alterations induced by HFD and the restorative effects of synergistic EGCG–taurine intervention. HFD significantly upregulated N-linoleyl proline and cortisol metabolites, confirming established lipotoxic pathways. EGCG intervention demonstrated characteristic elevation of polyphenolic compounds, consistent with its established antioxidant and lipid-lowering properties [33]. Taurine upregulated phospholipids and fluocinolone acetonide metabolites, reflecting its anti-inflammatory capacity and phospholipid metabolism regulation [34]. Combined EGCG–taurine treatment exhibited superior synergistic metabolic benefits compared to individual interventions. This combination preserved EGCG’s antioxidant properties and taurine’s anti-inflammatory effects while significantly reducing HFD-induced fatty acid synthesis. KEGG pathway enrichment analysis revealed that combined intervention established balanced metabolic homeostasis, characterized by significant enrichment of ABC transporters and coordinated activation of multiple vitamin metabolism pathways. This metabolic profile supported enhanced coenzyme function, indicating efficient substrate transport, moderate detoxification capacity, and balanced energy metabolism [45,46]. These findings demonstrate that EGCG–taurine combination effectively counteracts HFD-induced metabolic dysfunction through complementary antioxidant, anti-inflammatory, and metabolic regulatory mechanisms.

Our results suggest that the EGCG–taurine combination orchestrated metabolic reprogramming through the PPARα–FAS regulatory axis, which functions as a lipid sensor dynamically adjusting metabolic flux in response to fatty acid levels [61]. The observed PPARα activation was associated with promoted lipolysis and energy expenditure while suppressing FAS-mediated lipogenesis, which is consistent with established nutrient-sensing mechanisms [62]. Fox-o1 transcriptional activity determines metabolic allocation between energy storage and mobilization, serving as the master regulator of energy homeostasis across multiple tissues [63]. Tissue-specific LPL regulation provides local control over lipoprotein lipid uptake, enabling physiologically appropriate lipid distribution among metabolic tissues [64]. We propose that the synergistic effects of EGCG and taurine likely operate through complementary mechanisms targeting PPARα transcriptional activation and FAS post-translational modifications. EGCG enhanced PPARα-mediated fatty acid oxidation pathways, while taurine modulated FAS phosphorylation status, collectively achieving multi-target metabolic reprogramming. This coordinated intervention effectively reversed HFD-induced metabolic dysfunction by potentially simultaneously enhancing catabolic pathways and suppressing anabolic lipid synthesis.

In the present study, we selected exclusively male rats as experimental subjects based on well-established sex-related differences in metabolic physiology [65]. Compared to males, female subjects typically exhibit enhanced insulin sensitivity, distinct patterns of lipid storage and mobilization, and unique responses to metabolic perturbations. Estrogen profoundly influences multiple aspects of lipid homeostasis, including the promotion of hepatic lipogenesis, enhancement of lipoprotein lipase activity, and preferential distribution of adipose tissue to gluteofemoral depots. We are currently designing subsequent investigations that will include both male and female experimental animals, with appropriate consideration of estrous cycle phases in the experimental design. These follow-up studies will directly address whether the mechanisms identified in male subjects are conserved across sexes or whether sex-specific differences exist in the observed metabolic responses.

## 5. Conclusions

In summary, our comprehensive study demonstrates that the EGCG–taurine combination exerts enhanced metabolic effects against HFD-induced metabolic disorders through multiple interrelated mechanisms. This synergistic action is characterized by the following: (1) Directly modulating cellular lipid metabolism with greater efficacy in reducing TG accumulation compared to individual compounds; (2) comprehensive rebalancing of gut microbiota composition disrupted by adverse dietary patterns, notably normalizing the Firmicutes/Bacteroidetes ratio and enriching beneficial bacterial populations; (3) restoration of short-chain fatty acid production and intestinal barrier integrity, potentially establishing a foundation for improved host-microbiota metabolic crosstalk; (4) enhanced systemic lipid homeostasis via optimization of the PPARα/FAS signaling axis, directly addressing diet-induced metabolic dysregulation; and (5) global metabolic reprogramming toward homeostasis through simultaneous restoration of multiple pathways disrupted by high-fat dietary patterns. While monotherapies with EGCG or taurine showed partial efficacy, the EGCG–taurine combination enabled more comprehensive metabolic normalization by concurrently targeting the gut microbiota-mediated pathways that serve as critical mediators between dietary patterns and metabolic health. These findings provide preliminary scientific evidence that warrants further investigation into synergistic nutritional intervention strategies that leverage natural bioactive compounds to restore healthy dietary pattern–microbiota interactions, suggesting potential therapeutic approaches that require additional preclinical and clinical validation before translation to combating metabolic disorders associated with Western dietary patterns and related metabolic syndromes.

## Figures and Tables

**Table 1:** In vitro experimental protocol and treatment regimens for HepG2.

Groups	Treatment Protocol	Treatment Time
Control	High-glucose DMEM with 10% FBS	48 h total
OA	0.8 mmol/L oleic acid	48 h total
OA + EGCG	0.8 mmol/L oleic acid → 50 μg/mL EGCG	24 h + 24 h
OA + Taurine	0.8 mmol/L oleic acid → 100 μg/mL taurine	24 h + 24 h
OA + EGCG + Taurine	0.8 mmol/L oleic acid → 50 μg/mL EGCG + 100 μg/mL taurine	24 h + 24 h

**Table 2:** In vivo experimental protocol and treatment regimens for rats.

Groups	Oral Gavage	Dosage	Duration
NC	Saline	2 mL/100 g	8 weeks
MC	High-fat emulsion+ Saline	2 mL/100 g + 2 mL/100 g	8 weeks
PC	High-fat emulsion +Simvastatin	2 mL/100 g + 10 mg/kg	8 weeks
EC	High-fat emulsion + EGCG	2 mL/100 g + 100 mg/kg	8 weeks
TT	High-fat emulsion +Taurine	2 mL/100 g + 300 mg/kg	8 weeks
ETC	High-fat emulsion + (EGCG + Taurine)	2 mL/100 g + (100 mg/kg + 300 mg/kg)	8 weeks

**Table 3:** Effects of EGCG and taurine on rat serum parameters, lipid metabolism, hepatic function, and antioxidant status.

	NC	MC	PC	EC	TT	ETC
TG (mmol/L)	0.301 ± 0.045 ^d^	1.242 ± 0.284 ^a^	0.380 ± 0.047 ^cd^	0.455 ± 0.107 ^bc^	0.569 ± 0.106 ^b^	0.350 ± 0.030 ^d^
TC (mmol/L)	1.444 ± 0.137 ^d^	20.607 ± 1.307 ^a^	7.540 ± 1.750 ^c^	11.403 ± 1.767 ^bc^	13.070 ± 3.010 ^b^	7.601 ± 0.571 ^c^
HDL-C (mmol/L)	1.151 ± 0.033 ^a^	0.567 ± 0.064 ^c^	0.526 ± 0.052 ^c^	0.789 ± 0.041 ^b^	0.601 ± 0.075 ^c^	0.904 ± 0.092 ^ab^
LDL-C (mmol/L)	0.546 ± 0.280 ^d^	16.416 ± 0.807 ^a^	6.162 ± 1.816 ^c^	10.154 ± 0.502 ^b^	9.721 ± 1.264 ^b^	6.879 ± 0.616 ^c^
ALP (King/unit/100 mL)	34.41 ± 7.63 ^e^	139.51 ± 15.15 ^a^	81.37 ± 14.32 ^c^	96.59 ± 4.49 ^bc^	110.12 ± 2.96 ^b^	68.19 ± 2.89 ^cd^
ACC (μmol/h/g)	370.43 ± 17.05 ^b^	500.90 ± 12.77 ^a^	332.23 ± 14.97 ^b^	418.69 ± 20.47 ^ab^	448.64 ± 12.37 ^ab^	322.30 ± 11.76 ^b^
FAS (ng/mL)	35.44 ± 0.74 ^c^	65.27 ± 3.61 ^a^	41.62 ± 1.92 ^c^	50.97 ± 1.37 ^b^	50.06 ± 1.67 ^b^	42.37 ± 1.09 ^c^
LPL (μmol/min/mL)	137.19 ± 18.45 ^a^	2.35 ± 0.74 ^e^	102.34 ± 6.24 ^b^	49.46 ± 8.18 ^c^	19.61 ± 7.89 ^d^	75.61 ± 12.08 ^b^
FFA (μmol/mL)	69.94 ± 1.51 ^d^	124.22 ± 3.55 ^a^	74.69 ± 1.70 ^d^	82.07 ± 2.23 ^c^	87.13 ± 1.25 ^b^	71.46 ± 1.70 ^d^
ALT (nmol/min/mL)	29.60 ± 4.71 ^c^	110.65 ± 28.07 ^a^	64.56 ± 3.76 ^b^	63.15 ± 7.95 ^b^	68.13 ± 3.02 ^b^	31.02 ± 3.62 ^c^
AST (nmol/min/mL)	38.22 ± 2.77 ^c^	58.75 ± 3.15 ^a^	47.48 ± 2.64 ^b^	46.86 ± 1.94 ^b^	50.07 ± 1.31 ^b^	38.34 ± 0.53 ^c^
SOD (U/mL)	42.01 ± 1.70 ^a^	20.38 ± 2.61 ^c^	24.54 ± 0.67 ^bc^	27.96 ± 4.88 ^b^	30.68 ± 2.26 ^b^	36.82 ± 2.27 ^ab^
MDA (nmol/mL)	16.70 ± 3.30 ^c^	45.56 ± 5.75 ^a^	17.79 ± 3.42 ^c^	27.19 ± 1.62 ^b^	25.64 ± 2.67 ^b^	13.28 ± 0.85 ^c^
GSH-Px (U/mL)	5442.98 ± 260.47 ^a^	2673.26 ± 240.17 ^b^	4429.83 ± 219.90 ^ab^	3553.87 ± 307.67 ^b^	4665.75 ± 123.59 ^a^	5173.81 ± 185.46 ^a^

Data are means ± SEM (n = 3). a–e: Different letters in the same column indicate significant differences in the numerical values (*p* < 0.05). NC: normal diet control, MC: high-fat emulsion established model control, PC: simvastatin treatment positive control, EC: EGCG treatment, TT: taurine treatment, ETC: combined EGCG and taurine treatment.

**Table 4:** Intestinal microbial alpha diversity indices of rats after dietary treatment in different groups.

Groups	OUTs	ACE	Chao1	Simpson	Shannon
NC	759.33 ± 11.02 ^a^	763.35 ± 10.20 ^a^	759.85 ± 10.74 ^a^	0.97 ± 0.00097 ^a^	6.54 ± 0.027 ^a^
MC	419.67 ± 3.51 ^e^	426.95 ± 2.30 ^d^	422.80 ± 3.03 ^d^	0.96 ± 0.00050 ^c^	5.92 ± 0.017 ^c^
PC	610.33 ± 28.57 ^b^	614.23 ± 26.80 ^b^	610.71 ± 28.28 ^b^	0.98 ± 0.00040 ^a^	6.68 ± 0.030 ^a^
EC	547.33 ± 35.57 ^c^	539.10 ± 2.5 ^c^	548.11 ± 31.06 ^c^	0.97 ± 0.00061 ^b^	6.24 ± 0.011 ^b^
TT	510.67 ± 18.77 ^d^	513.23 ± 11.61 ^c^	512.45 ± 18.58 ^c^	0.97 ± 0.0006 ^b^	6.31 ± 0.028 ^b^
ETC	568.33 ± 25.42 ^bc^	589.65 ± 5.10 ^b^	568.82 ± 31.48 ^c^	0.98 ± 0.00045 ^a^	6.47 ± 0.035 ^ab^

Data are means ± SEM (n = 3). a–e: Different letters in the same column indicate significant differences in the numerical values (*p* < 0.05). NC: normal diet control, MC: high-fat emulsion established model control, PC: simvastatin treatment positive control, EC: EGCG treatment, TT: taurine treatment, ETC: combined EGCG and taurine treatment.

**Table 5:** Short-chain fatty acid levels in the feces of rats from different groups.

Group	Acetic Acid (μg/g)	Propionic Acid (μg/g)	Isobutyric Acid (μg/g)	Butyric Acid (μg/g)	Isovaleric Acid (μg/g)	Valeric Acid (μg/g)	Caproic Acid (μg/g)
NC	2640.73 ± 71.90 ^a^	426.69 ± 21.19 ^a^	440.33 ± 11.00 ^a^	274.92 ± 5.31 ^a^	42.74 ± 1.75 ^a^	269.87 ± 21.12 ^a^	420.49 ± 4.43 ^a^
MC	1336.67 ± 51.51 ^f^	220.37 ± 9.94 ^e^	239.89 ± 18.33 ^c^	197.90 ± 6.53 ^d^	25.14 ± 3.11 ^c^	196.50 ± 8.36 ^c^	284.94 ± 5.71 ^d^
PC	2250.17 ± 100.24 ^b^	404.98 ± 10.54 ^a^	425.29 ± 16.64 ^a^	253.99 ± 5.25 ^ab^	42.15 ± 1.06 ^a^	234.30 ± 3.85 ^b^	415.00 ± 5.12 ^a^
EC	1777.66 ± 70.70 ^d^	355.46 ± 11.58 ^c^	367.88 ± 2.89 ^b^	226.59 ± 4.19 ^bc^	33.03 ± 0.87 ^b^	217.45 ± 3.70 ^b^	345.14 ± 6.53 ^bc^
TT	1557.83 ± 91.45 ^e^	305.28 ± 10.43 ^d^	361.07 ± 1.62 ^b^	212.92 ± 6.80 ^c^	30.90 ± 0.28 ^b^	217.89 ± 13.43 ^b^	328.61 ± 8.17 ^c^
ETC	2051.56 ± 106.84 ^c^	385.75 ± 8.03 ^b^	399.96 ± 6.11 ^ab^	240.57 ± 7.59 ^b^	37.47 ± 2.05 ^ab^	236.10 ± 1.91 ^b^	369.91 ± 12.52 ^b^

Data are means ± SEM (n = 3). a–f: Different letters in the same row indicate significant differences in the numerical values (*p* < 0.05). NC: normal diet control, MC: high-fat emulsion established model control, PC: simvastatin treatment positive control, EC: EGCG treatment, TT: taurine treatment, ETC: combined EGCG and taurine treatment.

## Data Availability

The original Western blot data from this study are included in the Western blot full membrane images; for further inquiries, please contact the corresponding authors.

## Supplementary Materials

The following supporting information can be downloaded at: https://www.mdpi.com/article/10.3390/nu17162595/s1, Figure S1: Diagram illustrating the experimental groupings of animals; Figure S2: Body weight changes of rats in different groups from week 1 to 8; Figure S3: Effects of EGCG and taurine on rat gut microbiota; Figure S4: OPLS-DA score plots and permutation test plots across comparative groups; Figure S5: Principal component analysis and polar feature significance diagram. Figure S6: Metabolomics analysis.

## Abbreviations

The following abbreviations are used in this manuscript:

EGCG	Epigallocatechin gallate
TG	Triglyceride
TC	Total cholesterol
LDL-C	Low-density lipoprotein cholesterol
HDL-C	High-density lipoprotein cholesterol
SCFA	Short-chain fatty acid
PPARα	Peroxisome proliferator-activated receptor alpha
FAS	Fatty acid synthase
AMPK	AMP-activated protein kinase
PEPCK	Phosphoenolpyruvate carboxykinase
ACC	Acetyl-CoA carboxylase
SOD	Superoxide dismutase
GSH-Px	Glutathione peroxidase
DPPH	2,2-Diphenyl-1-picrylhydrazyl
MDA	Malondialdehyde
LPL	Lipoprotein lipase
FFA	Free fatty acid
NF-κB	Nuclear factor kappa-light-chain-Enhancer of activated B cells
Nrf2	Nuclear factor erythroid 2-related factor 2
Fox-o1	Forkhead box protein o1
HepG2	Human hepatoblastoma G2 cell line
DMEM	Dulbecco’s Modified Eagle Medium
FBS	Fetal bovine serum
ALP	Alkaline phosphatase
ALT	Alanine aminotransferase
AST	Aspartate aminotransferase
CCK-8	Cell counting kit-8
OA	Oleic acid
SEM	standard error of the mean
PMSF	Phenyl methyl sulfonyl fluoride
SPF	Specific pathogen-free
SD	Sprague Dawley
SDS-PAGE	Sulfate-polyacrylamide gel electrophoresis
OTU	Operational taxonomic unit
HFD	High-fat diet
PCA	Principal component analysis
PCoA	Principal coordinates analysis
OPLS-DA	Orthogonal partial least squares discriminant analysis
KEGG	Kyoto encyclopedia of genes and genomes

## References

[1] Chew N.W.S., Ng C.H., Tan D.J.H., Kong G., Lin C.X., Chin Y.H., Lim W.H., Huang D.Q., Quek J., Fu C.E. (2023). The global burden of metabolic disease: Data from 2000 to 2019. Cell Metab..

[2] Al Hroob A.M., Abukhalil M.H., Hussein O.E., Mahmoud A.M. (2019). Pathophysiological mechanisms of diabetic cardiomyopathy and the therapeutic potential of epigallocatechin-3-gallate. Biomed. Pharmacother..

[3] Zhao D.D., Zhang X.Z., Bian Y.X., Meng L., Wu Y.T., Ma Y.D., Li C., Wang J.J., Fu Z.Z., Dai J.Y. (2023). Taurine reduces apoptosis mediated by endoplasmic reticulum stress in islet 13-cells induced by high-fat and-glucose diets. Food Chem. Toxicol..

[4] Bellia A., Rizza S., Galli A., Fabiano R., Rossi R., Federici M., Sbraccia P., Lauro D. (2008). 12 Vascular and metabolic acute effects of rosuvastatin compared to simvastatin in untreated dyslipidemic diabetic patients. Nutr. Metab. Cardiovasc. Dis..

[5] Hursel R., Westerterp-Plantenga M.S. (2009). Green tea catechin plus caffeine supplementation to a high-protein diet has no additional effect on body weight maintenance after weight loss. Am. J. Clin. Nutr..

[6] Lee J.E., Min S.G., Hong S.W., Kim J.H., Kim G.H., Yun Y.R. (2023). Anti-obesity effects of kimchi with red yeast rice in 3T3-L1 adipocytes and high-fat diet-induced obese mice. J. Funct. Foods.

[7] Bao Y.F., Xiao J.B., Weng Z.B., Lu X.Y., Shen X.C., Wang F. (2020). A phenolic glycoside from *Moringa oleifera* Lam. improves the carbohydrate and lipid metabolisms through AMPK in *db*/*db* mice. Food Chem..

[8] Lopaschuk G.D., Ussher J.R., Folmes C.D.L., Jaswal J.S., Stanley W.C. (2010). Myocardial fatty acid metabolism in health and disease. Physiol. Rev..

[9] Amiot M.J., Riva C., Vinet A. (2016). Effects of dietary polyphenols on metabolic syndrome features in humans: A systematic review. Obes. Rev..

[10] Wang S.Z., Yu Y.J., Adeli K. (2020). Role of gut microbiota in neuroendocrine regulation of carbohydrate and lipid metabolism via the microbiota-gut-brain-liver axis. Microorganisms.

[11] Aravind S.M., Wichienchot S., Tsao R., Ramakrishnan S., Chakkaravarthi S. (2021). Role of dietary polyphenols on gut microbiota, their metabolites and health benefits. Food Res. Int..

[12] Liu Z.W., Ren Z.P., Zhang J., Chuang C.C., Kandaswamy E., Zhou T.Y., Zuo L. (2018). Role of ROS and nutritional antioxidants in human diseases. Front. Physiol..

[13] Bose M., Lambert J.D., Ju J., Reuhl K.R., Shapses S.A., Yang C.S. (2008). The major green tea polyphenol, (-)-epigallocatechin-3-gallate, inhibits obesity, metabolic syndrome, and fatty liver disease in high-fat-fed mice. J. Nutr..

[14] Cabrera C., Artacho R., Giménez R. (2006). Beneficial effects of green tea: A review. J. Am. Coll. Nutr..

[15] Seo D.B., Jeong H.W., Cho D., Lee B.J., Lee J.H., Choi J.Y., Bae I.H., Lee S.J. (2015). Fermented green tea extract alleviates obesity and related complications and alters gut microbiota composition in diet-induced obese mice. J. Med. Food.

[16] Yoon J.A., Shin K.O., Choi K.S. (2015). Studies on the function of Tturine: Review. Korean J. Food Nutr..

[17] Spady D.K., Turley S.D., Dietschy J.M. (1985). Rates of low density lipoprotein uptake and cholesterol synthesis are regulated independently in the liver. J. Lipid Res..

[18] Guo X.Z., Ou T., Yang X.Y., Song Q., Zhu L., Mi S.Q., Zhang J., Zhang Y.Z., Chen W., Guo J.X. (2024). Untargeted metabolomics based on ultra-high performance liquid chromatography-mass spectrometry/MS reveals the lipid-lowering mechanism of taurine in hyperlipidemia mice. Front. Nutr..

[19] Ahmadi S., Wang S.H., Nagpal R., Wang B., Jain S., Razazan A., Mishra S.P., Zhu X.W., Wang Z., Kavanagh K. (2020). A human-origin probiotic cocktail ameliorates aging-related leaky gut and inflammation via modulating the microbiota/taurine/tight junction axis. JCI Insight.

[20] Ahmed K., Choi H., Park J.S., Kim Y.G., Yim J.E. (2024). Taurine supplementation alters gene expression profiles in white adipose tissue of obese C57BL/6J mice: Inflammation and lipid synthesis perspectives. Heliyon.

[21] Wen J.J., Li M.Z., Chen C.H., Hong T., Yang J.R., Huang X.J., Geng F., Hu J.L., Nie S.P. (2022). Tea polyphenol and epigallocatechin gallate ameliorate hyperlipidemia via regulating liver metabolism and remodeling gut microbiota. Food Chem..

[22] Flint H.J., Scott K.P., Louis P., Duncan S.H. (2012). The role of the gut microbiota in nutrition and health. Nat. Rev. Gastroenterol. Hepatol..

[23] Wu J.Y., Wang K., Wang X.M., Pang Y.L., Jiang C.T. (2021). The role of the gut microbiome and its metabolites in metabolic diseases. Protein Cell..

[24] Cui W., Chen S.L., Hu K.-Q. (2010). Quantification and mechanisms of oleic acid-induced steatosis in HepG2 cells. Am. J. Transl. Res..

[25] Chen H., Boutros P.C. (2011). VennDiagram: A package for the generation of highly-customizable Venn and Euler diagrams in R. BMC Bioinf..

[26] Xie J.Y., Sun N., Huang H.R., Xie J.H., Chen Y., Hu X.B., Hu X.Y., Dong R.H., Yu Q. (2022). Catabolism of polyphenols released from mung bean coat and its effects on gut microbiota during in vitro simulated digestion and colonic fermentation. Food Chem..

[27] Lisec J., Schauer N., Kopka J., Willmitzer L., Fernie A.R. (2006). Gas chromatography mass spectrometry-based metabolite profiling in plants. Nat. Protoc..

[28] Wang J.L., Zhang T., Shen X.T., Liu J., Zhao D.L., Sun Y.W., Wang L., Liu Y.J., Gong X.Y., Liu Y.X. (2016). Serum metabolomics for early diagnosis of esophageal squamous cell carcinoma by UHPLC-QTOF/MS. Metabolomics.

[29] Zhuang B.Y., Hu F.C., Gao X., Leng Q., Zhang Y., You Y. (2025). Development of a simvastatin-loaded copolymer acid-sensitive nanocarrier and its experimental use in the treatment of keloids. J. Cosmet. Dermatol..

[30] Looft T., Johnson T.A., Allen H.K., Bayles D.O., Alt D.P., Stedtfeld R.D., Sul W.J., Stedtfeld T.M., Chai B.L., Cole J.R. (2012). In-feed antibiotic effects on the swine intestinal microbiome. Proc. Natl. Acad. Sci. USA.

[31] Amalia R., Pramono A., Afifah D.N., Noer E.R., Muniroh M., Kumoro A.C. (2022). Mangrove fruit (*Bruguiera gymnorhiza*) increases circulating GLP-1 and PYY, modulates lipid profiles, and reduces systemic inflammation by improving SCFA levels in obese wistar rats. Heliyon.

[32] Gou X.J., Feng Q., Fan L.L., Zhu J., Hu Y.Y. (2017). Serum and liver tissue metabonomic study on fatty liver in rats induced by high-fat diet and intervention effects of traditional Chinese medicine Qushi Huayu decoction. Evid. Based Complement. Altern. Med..

[33] Huang J., Wang Y., Xie Z., Zhou Y., Zhang Y., Wan X. (2014). The anti-obesity effects of green tea in human intervention and basic molecular studies. Eur. J. Clin. Nutr..

[34] Devi K.P.A., Martin A. (2022). Recent insights into the molecular regulators and mechanisms of taurine to modulate lipid metabolism: A review. Crit. Rev. Food Sci. Nutr..

[35] Tzang C.C., Chi L.Y., Lin L.H., Lin T.Y., Chang K.V., Wu W.T., Özcakar L. (2024). Taurine reduces the risk for metabolic syndrome: A systematic review and meta-analysis of randomized controlled trials. Nutr. Diabetes.

[36] Tang G.Y., Xu Y., Zhang C., Wang N., Li H.B., Feng Y.B. (2021). Green tea and epigallocatechin gallate (EGCG) for the management of nonalcoholic fatty liver diseases (NAFLD): Insights into the role of oxidative stress and antioxidant mechanism. Antioxidants.

[37] Jia W., Wei M.L., Rajani C., Zheng X.J. (2021). Targeting the alternative bile acid synthetic pathway for metabolic diseases. Protein Cell.

[38] Tu L.N., Showalter M.R., Cajka T., Fan S.L., Pillai V.V., Fiehn O., Selvaraj V. (2017). Metabolomic characteristics of cholesterol-induced non-obese nonalcoholic fatty liver disease in mice. Sci. Rep..

[39] Ye F., Dong M.C., Xu C.X., Jiang N., Chang Q., Liu X.M., Pan R.L. (2024). Effects of different chronic restraint stress periods on anxiety-and depression-like behaviors and tryptophan-kynurenine metabolism along the brain-gut axis in C57BL/6N mice. Eur. J. Pharmacol..

[40] Smeriglio A., Marcoccia D., Denaro M., Trombetta D. (2023). Nutraceuticals in the treatment of inflammatory bowel disease: How the panorama has changed in the last decade?. Curr. Med. Chem..

[41] Xin X., Cheng C., Cai B.Y., Li H.S., Tian H.J., Wang X., An Z.M., Sun Q.M., Hu Y.Y., Feng Q. (2021). Caffeine and EGCG alleviate high-trans fatty acid and high-carbohydrate diet-induced NASH in mice: Commonality and specificity. Front. Nutr..

[42] Xu Z., Kong X.Q. (2024). Bixin ameliorates high fat diet-induced cardiac injury in mice through inflammation and oxidative stress suppression. Biomed. Pharmacother..

[43] Delkhosh A., Shoorei H., Niazi V., Delashoub M., Gharamaleki M.N., Ahani-Nahayati M., Dehaghi Y.K., Raza S.H.A., Taheri M.M.H., Mohaqiq M. (2021). Coenzyme Q10 ameliorates inflammation, oxidative stress, and testicular histopathology in rats exposed to heat stress. Hum. Exp. Toxicol..

[44] Jiao N., Baker S.S., Chapa-Rodriguez A., Liu W.S., Nugent C.A., Tsompana M., Mastrandrea L., Buck M., Baker R.D., Genco R.J. (2017). Suppressed hepatic bile acid signalling despite elevated production of primary and secondary bile acids in NAFLD. Gastroenterology.

[45] Dauchy S., Dutheil F., Weaver R.J., Chassoux F., Daumas-Duport C., Couraud P.O., Scherrmann J.M., De Waziers I., Declèves X. (2008). ABC transporters, cytochromes P450 and their main transcription factors: Expression at the human blood-brain barrier. J. Neurochem..

[46] Velazquez-Arellano A., Hernandez-Vazquez A. (2020). Vitamins as cofactors for energy homeostasis and their genomic control, with special reference to biotin, thiamine, and pantothenic acid. Princ. Nutr. Nutr..

[47] Cheng Z.Y., White M.F. (2010). Foxo1 in hepatic lipid metabolism. Cell Cycle.

[48] Peeters A., Baes M. (2010). Role of PPARα in hepatic carbohydrate metabolism. PPAR Res..

[49] Moszak M., Szulinska M., Bogdanski P. (2020). You are what you eat-the relationship between diet, microbiota, and metabolic disorders-a review. Nutrients.

[50] Galassi A., Reynolds K., He J. (2006). Metabolic syndrome and risk of cardiovascular disease: A meta-analysis. Am. J. Med..

[51] Gai Z.B., Wang T.Q., Visentin M., Kullak-Ublick G.A., Fu X.J., Wang Z. (2019). Lipid accumulation and chronic kidney disease. Nutrients.

[52] Soussi A., Gargouri M., Magné C., Ben-Nasr H., Kausar M.A., Siddiqui A.J., Saeed M., Snoussi M., Adnan M., El-Feki A. (2022). (-)-Epigallocatechin gallate (EGCG) pharmacokinetics and molecular interactions towards amelioration of hyperglycemia, hyperlipidemia associated hepatorenal oxidative injury in alloxan induced diabetic mice. Chem. Biol. Interact..

[53] Wang T.Q., Fu X.J., Chen Q.F., Patra J.K., Wang D.D., Wang Z.G., Gai Z.B. (2019). Arachidonic acid metabolism and kidney inflammation. Int. J. Mol. Sci..

[54] Chiang J.Y.L., Ferrell J.M., Wu Y., Boehme S. (2020). Bile acid and cholesterol metabolism in atherosclerotic cardiovascular disease and therapy. Cardiol. Plus.

[55] Samuel V.T., Shulman G.I. (2018). Nonalcoholic fatty liver disease as a nexus of metabolic and hepatic diseases. Cell Metab..

[56] Zhu W.H., Chen S.W., Chen R.G., Peng Z.Q., Wan J., Wu B.Y. (2017). Taurine and tea polyphenols combination ameliorate nonalcoholic steatohepatitis in rats. BMC Complement. Altern. Med..

[57] Mukhopadhya I., Louis P. (2025). Gut microbiota-derived short-chain fatty acids and their role in human health and disease. Nat. Rev. Microbiol..

[58] Magne F., Gotteland M., Gauthier L., Zazueta A., Pesoa S., Navarrete P., Balamurugan R. (2020). The firmicutes/bacteroidetes ratio: A relevant marker of gut dysbiosis in obese patients?. Nutrients.

[59] Liu X.X., Zhao K., Jing N.N., Zhao Y., Yang X.B. (2020). EGCG regulates fatty acid metabolism of high-fat diet-fed mice in association with enrichment of gut *Akkermansia muciniphila*. J. Funct. Foods.

[60] Qian W.K., Li M.Y., Yu L.L., Tian F.W., Zhao J.X., Zhai Q.X. (2023). Effects of taurine on gut microbiota homeostasis: An evaluation based on two models of gut dysbiosis. Biomedicines.

[61] Pawlak M., Lefebvre P., Staels B. (2015). Molecular mechanism of PPARα action and its impact on lipid metabolism, inflammation and fibrosis in non-alcoholic fatty liver disease. J. Hepatol..

[62] Geng X.Y., Feng Y.F., Yu C.C., Yao Y.Y., Chen W., Guo J.X., Zhang Y.Z., Zhang J., Mi S.Q. (2024). Taurine supplementation decreases fat accumulation by suppressing FAS and enhancing ATGL through the ATGL pathway. Iran. J. Basic Med. Sci..

[63] Peng S.M., Li W., Hou N.N., Huang N. (2020). A review of *FoxO1*-regulated metabolic diseases and related drug discoveries. Cells.

[64] Wang H., Eckel R.H. (2009). Lipoprotein lipase: From gene to obesity. Am. J. Physiol..

[65] Tramunt B., Smati S., Grandgeorge N., Lenfant F., Arnal J.F., Montagner A., Gourdy P. (2020). Sex differences in metabolic regulation and diabetes susceptibility. Diabetologia.

