# Supplementary material for: EGCG and Taurine Synergistically Ameliorate Lipid Metabolism Disorder by Modulating Gut Microbiota and PPARα/FAS Signaling Pathway
Source: Nutrients. 2025 Aug 9;17(16):2595. doi: 10.3390/nu17162595 (PMC12389005; doi:10.3390/nu17162595)
Supplement: Supplementary file 1 [file nutrients-17-02595-s001.zip › nutrients-3770115-supplementary.pdf]

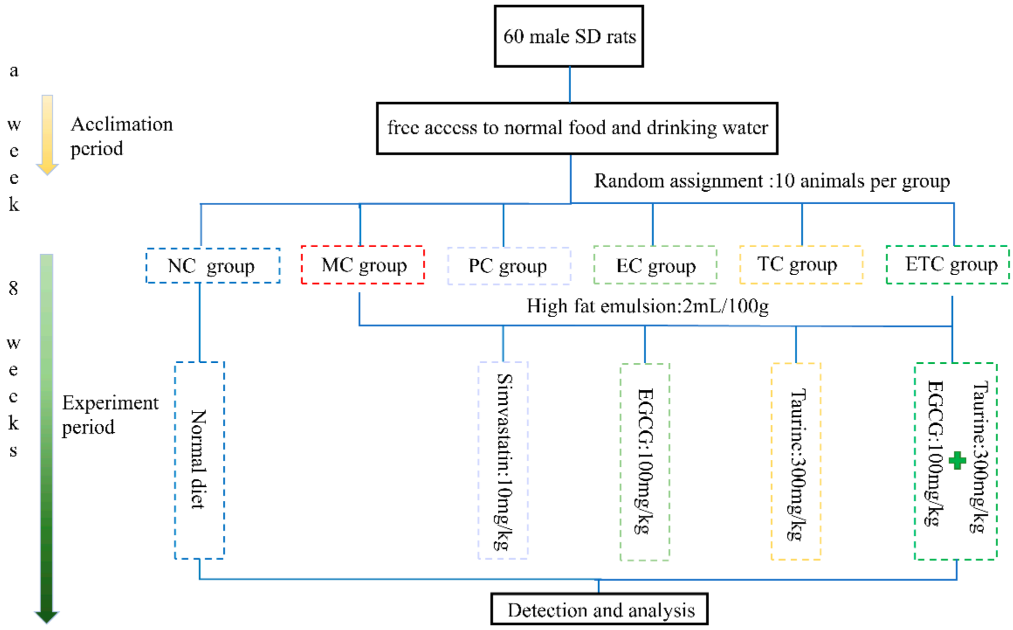

Figure S1: Diagram illustrating the experimental groupings of animals. Normal control group (NC), normal diet feed; Model control group (MC), high fat diet feed; EGCG (EC), EGCG (100 mg/kg); Taurine (TC), taurine (300 mg/kg); EGCG and taurine synergy group (ETC), EGCG (100 mg/kg) and taurine (300 mg/kg); and positive control group (PC), simvastatin (10 mg/kg).

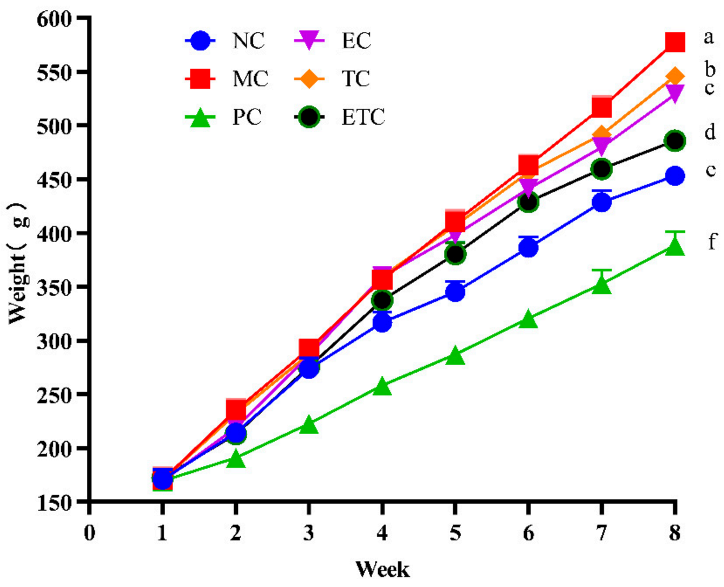

Figure S2: Body weight changes of rats in different groups from week 1 to 8. a-f: Different letters in the same row indicate significant differences in the numerical values ($p < 0.05$). NC: Normal control group; MC: Model control group; EC: EGCG group; TC: Taurine group; ETC: EGCG and taurine synergy group.

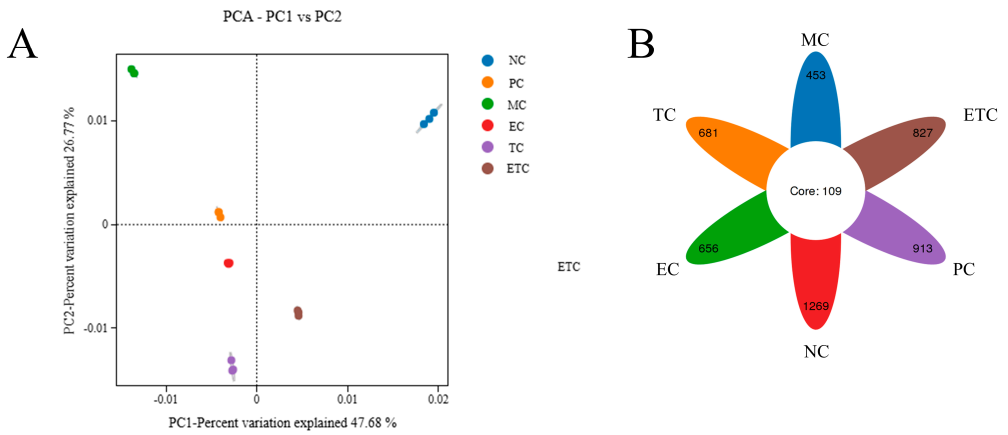

Figure S3. Effects of EGCG and taurine on rat gut microbiota. A: PCA analysis; B: Flower diagram. NC: Normal control group; MC: Model control group; EC: EGCG group; TC: Taurine group; ETC: EGCG and taurine synergy group.

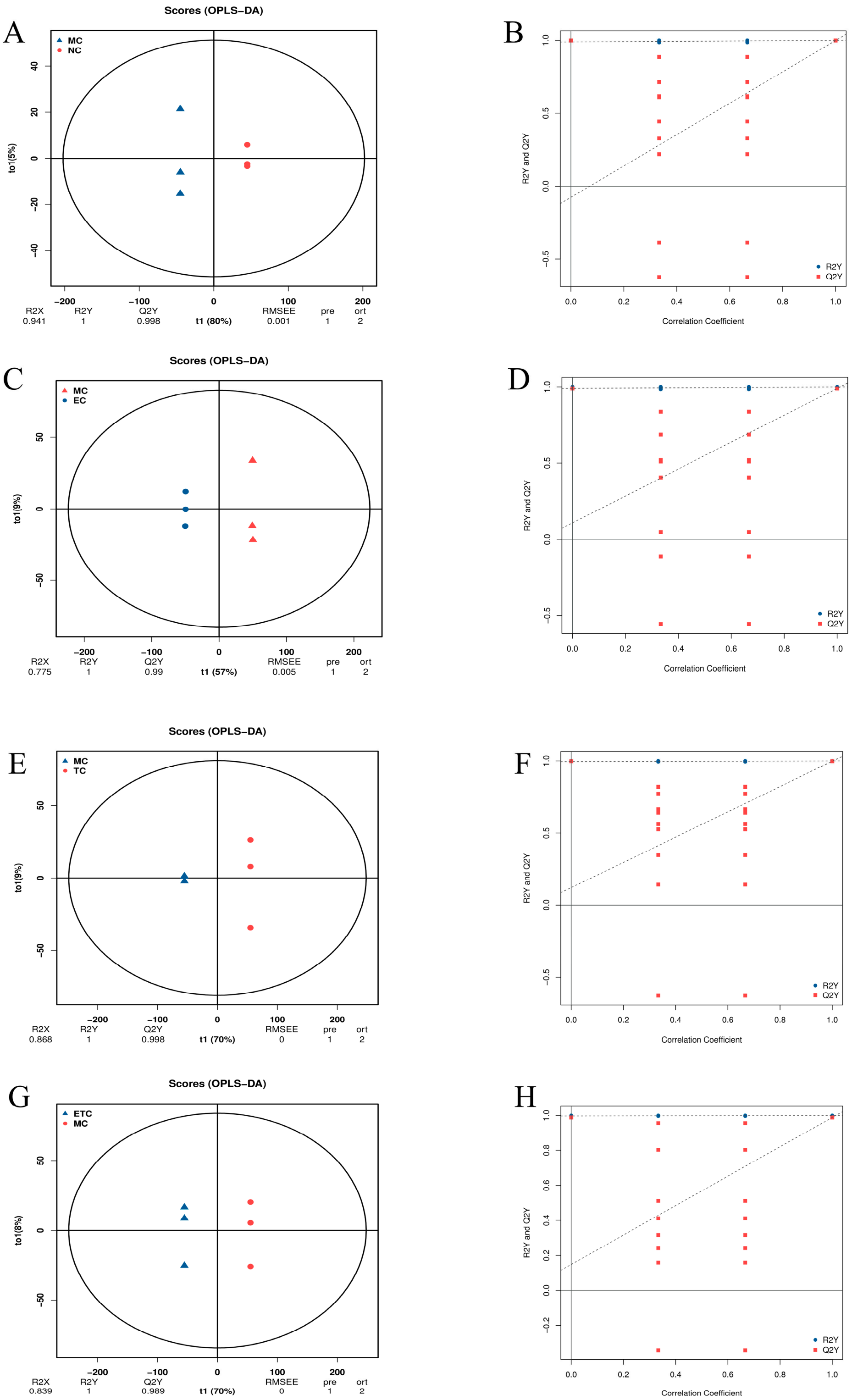

Figure S4: OPLS-DA score plots and permutation test plots across comparative groups. A：MC vs NC OPLS-DA score plots; B：MC vs NC OPLS-DA permutation test plots；C：EC vs MC OPLS-DA score plots；D：EC vs MC OPLS-DA permutation test plots；E：TC vs MC OPLS-DA score plots；F：TC vs MC OPLS-DA permutation test plots；G：ETC vs MC OPLS-DA score plots；H：ETC vs MC OPLS-DA permutation test plots. NC: Normal control group; MC: Model control group; EC: EGCG group; TC: Taurine group; ETC: EGCG and taurine synergy group.

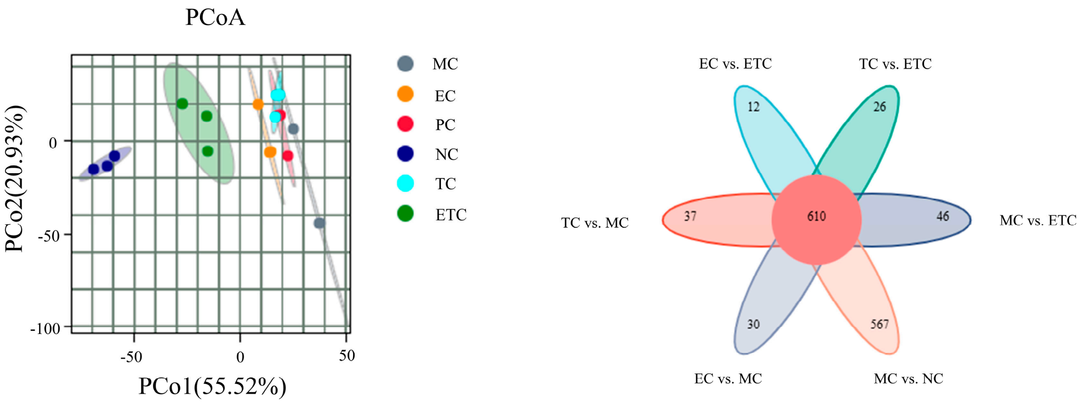

A B

Figure S5: Principal coordinate analysis and polar feature significance diagram. A: Principal coordinate analysis; B: Flower diagram. NC: Normal control group; MC: Model control group; EC: EGCG group; TC: Taurine group; ETC: EGCG and taurine synergy group.

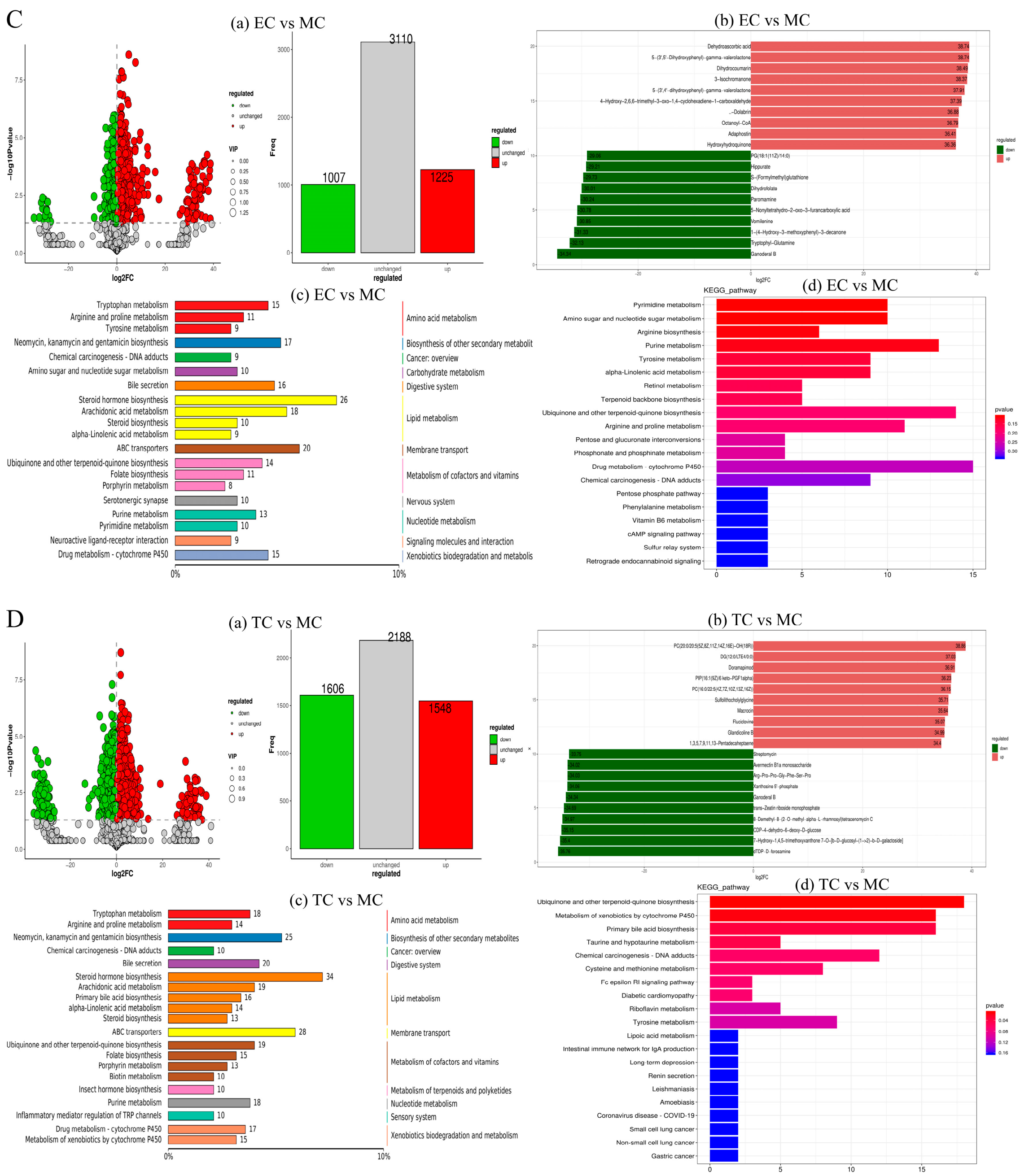

Figure S6: Metabolomics analysis. A：EC vs MC；B：TC vs MC. (a-d): Volcano plot, differential fold-change bar chart, classification of differential metabolite pathways, and KEGG pathway bar chart. MC: Model control group; EC: EGCG group; TC: Taurine group.
